# Developing recommendations on ethical aspects affecting studies in health professions education research

**DOI:** 10.3205/zma001795

**Published:** 2026-01-15

**Authors:** Johanna Hirsch, Marianne Giesler, Jan Matthes, Angelika Homberg, Monika Himmelbauer, Daniel Bauer, Martin Boeker, Katrin Schüttpelz-Brauns

**Affiliations:** 1 Heidelberg University, Medical Faculty Mannheim, Mannheim, Germany; 2 Freiburg i. Br., Germany; 3 University of Cologne, Faculty of Medicine, Student Dean’s Office, Cologne, Germany; 4 University of Cologne, Faculty of Medicine and University Hospital, Center for Pharmacology, Cologne, Germany; 5 Medical University of Vienna, Teaching Center, Vienna, Austria; 6 University of Bern, Institute for Medical Education, Bern, Switzerland; 7 Technical University of Munich, TUM University Hospital, Munich, Germany

**Keywords:** ethics research, health professions education research, medical education research

## Abstract

**Background:**

In addition to meeting legal requirements, education research studies in the health professions must also factor in aspects of ethical research. However, no specific guidance exists as of yet on how to implement these requirements and aspects in practice. Our aim was therefore to develop recommendations on how to apply aspects of ethical research when planning, conducting and publishing such studies.

**Method:**

The recommendations were developed by the members of the *DACH Association for Medical Education’s Committee on Methodology in Educational Research* in three steps: (1) identification of problematic areas in education research, (2) derivation of concrete measures and explanations, and (3) review of the recommendations by experts with different research perspectives.

**Results:**

Two separate sets of recommendations were developed. One set contains actions to ensure data protection, free consent, no harm to and the autonomy of participants (six areas with a total of 29 measures); the other addresses the quality assurance of studies and publications (four areas with a total of 14 measures).

**Discussion:**

The recommendations provide assistance when considering ethical aspects and applying them. However, they do not replace any existing rules or the individual reflections of the researchers. These recommendations should undergo continual development and be investigated in regard to their application and effect on decision-making processes in education research.

## Zusammenfassung

**Hintergrund:**

Studien zur Ausbildungsforschung in den Gesundheitsberufen müssen neben gesetzlichen Vorgaben auch forschungsethische Aspekte berücksichtigen. Eine spezifische Orientierungshilfe, um diese Vorgaben und Aspekte in die Praxis umzusetzen, fehlt jedoch bislang. Unser Ziel war es daher, Handlungsempfehlungen für die Berücksichtigung forschungsethischer Aspekte bei der Planung, Durchführung und Veröffentlichung derartiger Studien zu entwickeln.

**Methode:**

Die Handlungsempfehlungen wurden durch Mitglieder des *Ausschusses für Methodik der Ausbildungsforschung der Gesellschaft für Medizinische Ausbildung* in drei Schritten erstellt: (1) Erarbeitung von Problemfeldern in der Ethik der Ausbildungsforschung, (2) Ableitung konkreter Maßnahmen und Erläuterungen, (3) Überprüfung der Handlungsempfehlungen durch Expert:innen verschiedener Forschungsperspektiven.

**Ergebnisse:**

Es wurden zwei separate Handlungsempfehlungen erarbeitet, die Maßnahmen zur Sicherstellung des Datenschutzes, der Freiwilligkeit, Nicht-Schädigung und Selbstbestimmung von Teilnehmenden (sechs Bereiche mit insgesamt 29 Maßnahmen) sowie zur Qualitätssicherung der Studie und Veröffentlichung (vier Bereiche mit insgesamt 14 Maßnahmen) umfassen.

**Diskussion:**

Die Handlungsempfehlungen bieten eine Unterstützung zur Berücksichtigung ethischer Aspekte und deren Umsetzung. Sie ersetzen jedoch nicht bereits bestehende Regelungen sowie die individuelle Reflexion der Forschenden. Sie sollten kontinuierlich weiterentwickelt und in Bezug auf ihre Anwendung und Wirkung auf Entscheidungsprozesse in der Ausbildungsforschung untersucht werden.

## 1. Introduction

Aspects of ethical research must be taken into account, in addition to the applicable laws, when planning, conducting and publishing education research studies in the health professions. The key concepts in this paper may be construed differently. We use the terms as defined in table 1 (Tab. 1).

Various codes of conduct and guidelines from other fields are available to education researchers to reflect on ethical research principles and use as guidance in their work, e.g., the World Medical Association’s *Declaration of Helsinki* [1], the *Belmont Report* [2], the *Ethical Guidelines for Educational Research* of the *British Educational Research Association* (BERA) [3], and even institutional and national guidelines on good scientific practice, such as the German Research Foundation’s (DFG) *Code of Conduct: Guideline on Safeguarding Good Research Practice* [4]. Handbooks and publications in journals offer an opportunity to engage with research ethics and contain common examples on how the principles can be implemented in the research process, e. g. [5], [6], [7], [8]. Since the sources cited here were not developed for education research, education researchers must decide for themselves which of the aspects covered in them are relevant to their study and transfer them to their particular setting. They also must identify the aspects which are not addressed in the available sources, but which are nonetheless important for their study. For instance, the *Declaration of Helsinki* contains ethical principles for medical research involving humans [1]; however, there is no information about how to deal with students or trainees as study participants or how to develop relevant questions or hypotheses in the field of education research.

Communication between fellow researchers and shared insights within research teams can likewise be important in the adherence to principles of ethical research, for example, when no guidelines or practical information exist. Also helpful is when research teams have members who acquired knowledge concerning ethical research and proper conduct in the research process during their university studies and/or post-graduate training and are able to draw upon it.

Furthermore, there are a variety of academic papers in the field of health professions education research that deal with individual aspects of ethical research, e.g. [9], [10], [11].

Advice from an ethics committee can also contribute to the consideration and implementation of ethical aspects. Whether a review or evaluation by an ethical committee is necessary depends on different factors, such as the professional groups to which the researchers belong, the internal rules of the university faculty or institution with which the researchers are affiliated, and the source of funding for the study, e.g., external funding is usually contingent on formal ethical approval. However, advice or approval from an ethics committee is not offered or required at all institutions where education research studies are conducted. Moreover, as of yet not all local ethics committees have prepared themselves to evaluate education research studies so important ethical aspects may remain unaddressed in their evaluations or advice.

Regardless of any formal connection to an ethics committee, education researchers are individually responsible for taking ethical aspects into careful consideration and implementing them in practice over the entire research process. Despite the above possibilities, it is often challenging to find the time to consider the ethical issues involved in research in depth during regular working hours. Moreover, neither one-size-fits-all nor quick-and-easy solutions exist in research ethics because ethical decisions are always made individually and must be adapted to the given situation. What is particularly challenging is that for the specific field of education research there is, as of yet, no customized guidance linking the general principles of ethical research in human subjects with the specific methodological and content-based requirements of education research. This research gap creates uncertainty and impedes a uniform and practical application of ethical standards.

Against this backdrop, it makes sense to develop a tool for systematic consideration of ethical aspects in research during the entire research process in education research. Such a tool can play a role in the early recognition of and appropriate response to potential ethical challenges, as well as systematically and transparently describing how ethical standards are considered and observed.

Therefore, the aim is to develop recommendations for taking aspects of ethical research into account when planning, conducting and publishing studies in health professions education research.

## 2. Methodology

The recommendations were developed in three steps:

1. identification of problematic areas in education research,
2. derivation of concrete measures and explanations,
3. review of the recommendations by experts with different research perspectives.

The members of the ethics working group (Ethics WG) of the *DACH Association for Medical Education’s Committee on Methodology in Educational Research* participated in these steps in varying constellations and are the authors of this paper.

The three-step process was chosen to fully utilize all of the available sources, meaning publications, knowledge and experience-based expertise of the working group members, and the experts’ feedback. This was to ensure that the recommendations are as comprehensive, straightforward, and correctly worded as possible.

### 2.1. Identification of problematic areas

“Problematic areas” are understood here to mean topics where education researchers see themselves confronted with ethically relevant issues when planning and conducting studies. Each problematic area encompasses multiple aspects that describe the specific ethical issues connected with a topic.

An explorative literature review was carried out to identify these problematic areas and the aspects contained within them. The focus was on orientation in the topical area. The research was carried out in PubMed, ERIC, Fachportal Pädagogik, the Journal of Medical Ethics’ archive, and using Google and Google Scholar. The search terms were developed iteratively. Examples of the queries can be found in attachment 1 , table A1.

The selected publications address ethical issues relevant to research practice in health professions education research, have lead authors who are active in education research or adjacent fields, such as psychology or ethics/research ethics, and are available in German or English. No limitations were set in regard to type of publication, date or country of publication.

All of the text passages with information on ethical issues relevant to research practice in health professions education research were extracted from the selected publications, along with the general details of the papers. English texts were translated using the translation program DeepL^®^ pro version Q1/Q2 2024 (DeepL SE, Cologne).

The extracted passages were compiled and sorted according to topic. On the basis of this thematic categorization, it was possible to identify problematic areas. The aspects of ethical research addressed in the texts were then assigned to the corresponding problematic area and supplemented in terms of content. The content of the problematic areas is based on multiple publications.

### 2.2. Development of measures and explanations

Based on the identified problematic areas, the authors developed targeted measures regarding the ethical aspects for research. Online meetings were held for the individual problematic areas, whereby attendance varied from meeting to meeting (AH, DB, JH, JM, KSB, MB, MG, MH). A total of five online meetings lasting from two to four hours took place between February and May, 2024. Each time, five to seven people participated including the moderators (KSB and JH). At each meeting, the participants were randomly divided into two groups that independently worked on the same task according to a prescribed flowchart (see attachment 1 , figure A2). The results were then subsequently discussed in the full group and, where possible, a consensus was reached. If agreement on an aspect could not be found in the smaller groups or in the larger group or if a lack of content was identified, this was then noted and additional literature research was carried out, the publications already found were re-read, and the application forms used by ethics committees were reviewed and used as the basis for further discussions with all of the individuals participating in the process up to that time point.

The fully mapped out measures were checked for redundancies and then inserted into a table. The wording of the measures was condensed and additional information and explanations were listed in a separate column. The measures and their explanations were grouped together. To avoid misunderstandings in the application of the measures, they were revised in several rounds by four working group members (MG, JH, JM, KSB) until no further aspects were noted. Following this, the other working group members were able to add suggested changes. Thus, after another round of revision, the measures and their explanations existed in tabular form and, as such, formed the first version of the recommendations.

### 2.3. Verification of the recommendations by experts from different research perspectives

To check the recommendations for accuracy of content, comprehensibility and completeness, experts who had not been involved in generating the measures and explanations were identified and contacted, based on predefined selection criteria (see attachment 1 , figure A3), through internet research, or recommendation by an already contacted expert. After giving informed verbal consent, these experts received the recommendations along with instructions via email. Content falling outside of their area of expertise needed to be marked. This was necessary to determine whether all of the content was evaluated by experts. Two working group members integrated the experts’ feedback.

The recruited experts come from the fields of medical ethics, psychology, nursing science, general ethics and law. Four of the experts have experience in planning, conducting, evaluating and publishing studies as lead author or co-author. In addition, two of the experts are also well versed in qualitative research, and one person in medical research following different research methods. The person with legal expertise provided a perspective on the measures and explanations from the vantage point of legal analysis and advising on research projects, especially in regard to the issue of data protection within the scope of the *General Data Protection Regulation* (GDPR). Specialized knowledge in quantitative research had already been represented by three people working on the recommendations in the ethics WG, which is why no additional external experts were recruited for this. Among the external experts, two individuals have experience with teaching academic courses, two serve on ethics committees, and one is active in networking among ethics committees. According to the information provided by the experts, all of the measures listed in both sets of recommendations fell within the remit of at least one person.

The wording of the measures and explanations in both sets of recommendations was refined and stated more precisely based on the experts’ comments, missing information and definitions were inserted, and a measure was added to one set of recommendations.

## 3. Results

### 3.1. Identified problematic areas

A total of 45 publications were found in the explorative literature review and screening was undertaken from October 2023 to February 2024 (see attachment 1 , figure A4, for the selection process). These were used to identify the problematic areas and describe the individual ethical aspects in these areas.

Table 2 (Tab. 2) contains details on type and year of publication, journal, and lead author’s area of specialty at the time the original sources were published.

Six problematic areas were identified:

1. research methodology,
2. compliance with laws and guidelines,
3. good scientific practice in publications,
4. competence of ethics committees,
5. students as a vulnerable group,
6. dealing with other study populations.

Attachment 2 outlines the problematic areas with their associated aspects, along with the literature on which the content is based.

### 3.2. Final versions of the recommendations

As the measures were compiled, it became clear that individual measures could be assigned to more than one problematic area. Redundancies were identified through restructuring, and the measures then placed in new categories. These categories form the basis for the areas presented in the two sets of recommendations. As this process took place, two different points of focus came into view: first, the protection of study participants and individuals potentially affected by a study and, second, the quality of a study and its publication. These two focal points each function as main categories for the areas covered by them along with their associated measures. For this reason, the recommendations were drafted in two different sets, each as its own separate document.

The *Recommendations on ensuring Data Protection, Free Consent, No Harm to and Autonomy of Participants and Potentially Affected Individuals (Rec-Protect)* now covers six areas:

- observing data minimization in regard to personal data
- protecting participants’ personal data and sensitive personal data
- protecting the privacy and identity of study participants and individuals who decide against or terminate participation
- handling potential risks and stresses for study participants and individuals affected by the study
- ensuring informed and free decisions to participate or not in a study
- assuring independent ethical review and legal compliance in study planning and conduction

A total of 29 measures were assigned to these areas. Explanations were written for each measure and contain, e.g., definitions of ambiguous terms, reasons why a particular measure is important, and information on possible implementation. All of the recommendations concerning protection (Rec-Protect) are presented in tabular form in attachment 3 .

The *Recommendations on Assuring the Quality of a Study and its Publication (Rec-Quality*) covers four areas and has 14 measures.

The areas in this set of recommendations are:

- assuring good scientific practice
- complying with legal requirements and standard guidelines
- visibility of the study
- confirmability of the study.

All of the recommendations concerning study quality are presented in tabular form in attachment 4 .

The feedback from the experts also contained aspects that were missing from the recommendations or which had been given too little attention up to that point. These topics include: participative research approaches, collaborative relationships between researchers and study participants, responsibility for mentoring student researchers, delineating between evaluation and research, dealing with the pressure to publish, standards for peer review, conflicts of interest in reviewers, and retracting or censoring publications.

## 4. Discussion

As a result of the multi-step process, it is possible to provide comprehensive recommendations with measures to implement aspects of ethical research in the planning, conduction and publication of education research studies.

The literature review, the basis on which the problematic areas and some of the ethical research aspects in them were identified, was meant to be exploratory and not systematic. This may result in relevant literature being overlooked and thus the risk of gaps in the identification of problematic areas and the compilation of aspects pertaining to research ethics. A systematic literature review could have yielded a more comprehensive overview of relevant publications. That said, the aim here was not to take recommendations from the literature, but instead to develop measures and explanations based on the literature in an iterative discourse and then have them reviewed by external experts from multiple perspectives. Following this approach, we were unable to take into account future developments in research methodology affecting aspects of ethical research not yet considered.

The iterative group process consisted of the discussions during the meetings of the ethics WG of the *Committee on Methodology in Educational Research* and the written comments on the individual measures and explanations in the revision rounds. During the revision rounds, the participants had plenty of time to read the documents, formulate their assessments of the content and submit their suggestions for improvement in writing. This process enabled thorough scrutiny of the content and facilitated a detailed and deliberate revision. The challenge lay primarily in giving all of the comments adequate attention, which is why an iterative approach was necessary. A formal consensus procedure, such as the Delphi technique, to generate and agree upon the recommendations was not conducted because the sheer scope of the documents would have exceeded both the timeline and staff resources.

The two sets of recommendations are quite extensive in terms of content. Although the individual measures are concisely stated, the associated explanations, however, require more space to adequately present the complex connections and cover the different types of research and methods in health professions education research.

The range of the different studies in the field of education research is very large, so that not all of the aspects in the recommendations are relevant for each individual study and it may not always be possible to implement some aspects in the ways recommended. This can indicate moments for reflection and discussion in research teams or with experts in the relevant areas. It is also possible that points are brought up in the recommendations that contradict one's own attitudes, convictions and practices. Such a discrepancy can also provide the basis for reflection, discussion and sharing for the purpose of deciding which options are available for a planned study. Moreover, the recommendations should not be viewed as fixed and final, but rather as undergoing continual review and further development. In the course of this, the topics that were mentioned by the experts but have not yet been addressed should be given closer attention. These topics were either touched on only briefly or not yet dealt with when developing the present recommendations because, initially, the aim was to cover the basic aspects of ethical research.

Furthermore, updating these recommendations in the future offers the possibility to include international perspectives, e.g., on data protection, in more detail and elaborate on them in the measures and their explanations. Likewise, current trends such as Big Data analysis and learning analytics [12] can be addressed through revision. Also, the application and practicability of the recommendations should be investigated, particularly in regard to how they shape researchers' decision-making about ethical research aspects when planning, conducting and publishing studies.

These recommendations are not a replacement for but rather a supplementation of existing materials concerned with reflecting on and implementing principles of ethical research, such as ethical codes of conduct, e.g. [1], [2], [3], [4], scientific literature, e.g. [5], [6], [7], [8], [9], ethics committee opinions, collegial interchange, and compliance with legal requirements, e.g., data protection. It should also be noted that the recommendations were formulated from the local perspectives of the authors and external experts (DE, CH, AT). For instance, in regard to the topic of data protection, the measures and explanations have been composed based on the GDPR – which has a specific scope of validity – meaning that the recommendations should be viewed as an impetus to become informed about the rules and requirements applicable to a particular study and follow them accordingly. Nevertheless, we assume that, generally, the measures can be applied internationally and their implementation can be aligned with and adapted to local rules and regulations.

## Note

The following references were found through the literature review and included in the identification of problematic areas: [9], [10], [11], [13], [14], [15], [16], [17], [18], [19], [20], [21], [22], [23], [24], [25], [26], [27], [28], [29], [30], [31], [32], [33], [34], [35], [36], [37], [38], [39], [40], [41], [42], [43], [44], [45], [46], [47], [48], [49], [50], [51], [52], [53], [54].

## Acknowledgements

We wish to thank Dr. rer. biol. hum. Katja Kühlmeyer, Ass.-Prof. Ph.D. Christine Dunger, Dr. med. Sonja Mathes, Dr. phil. Matthias Katzer and Rebekka Kiser for their feedback on the recommendations.

## Authors’ ORCIDs

- Johanna Hirsch: [0009-0002-9818-5287]
- Marianne Giesler: [0000-0001-9384-2343]
- Jan Matthes: [0000-0003-2754-1555]
- Angelika Homberg: [0000-0001-5585-1126]
- Monika Himmelbauer: [0000-0001-5516-1993]
- Daniel Bauer: [0000-0002-3337-3327]
- Martin Boeker: [0000-0003-2972-2042]
- Katrin Schüttpelz-Brauns: [0000-0001-9004-0724]

## Competing interests

The authors declare that they have no competing interests. 

## Supplementary Material

Supplementary material

Problematic areas

Recommendations on ensuring data protection, free consent, no harm to and autonomy of par-ticipants and potentially affected individuals (rec-protect)

Recommendations on assuring the quality of a study and its publication (rec-quality)

## Figures and Tables

**Table 1:**
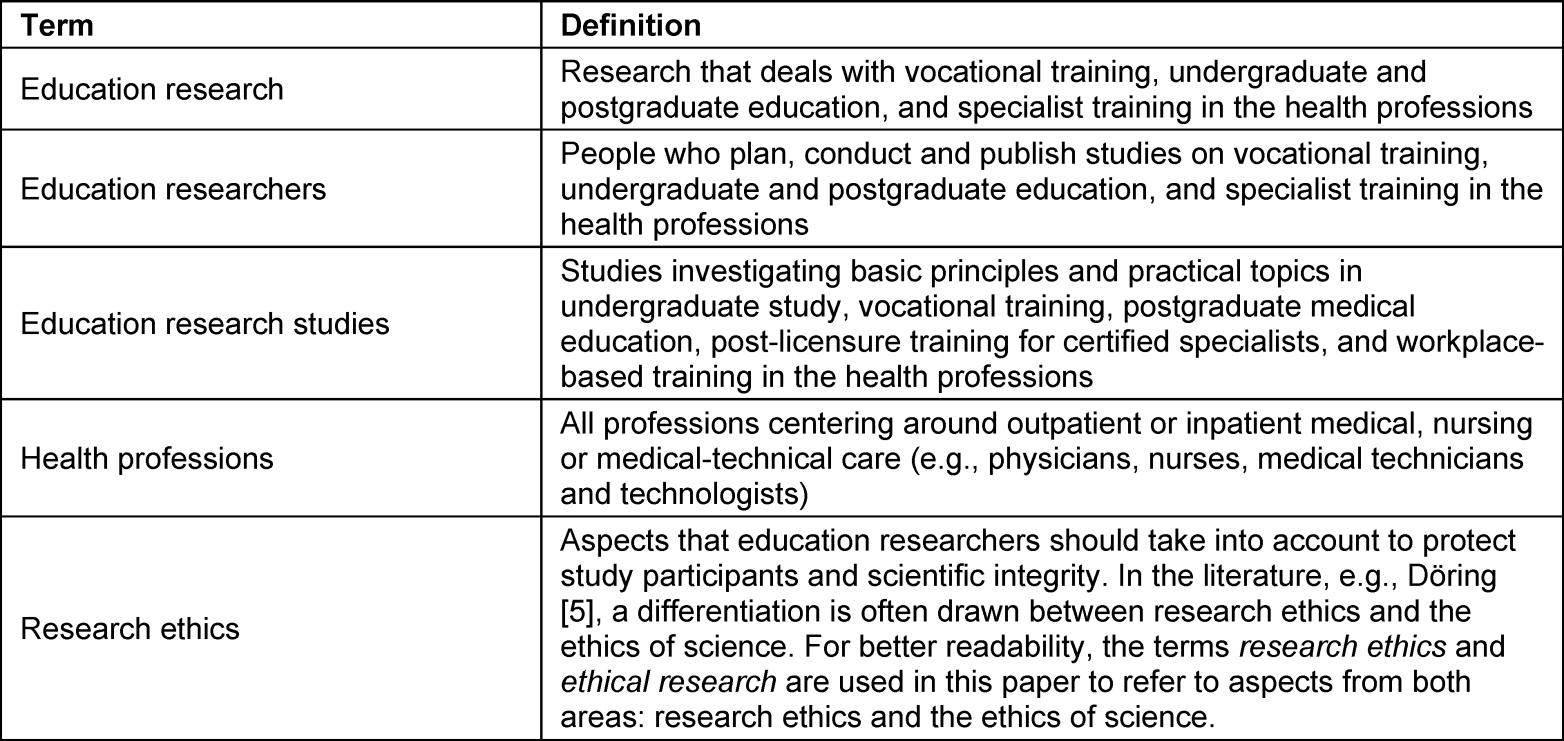
Glossary of key terms

**Table 2:**
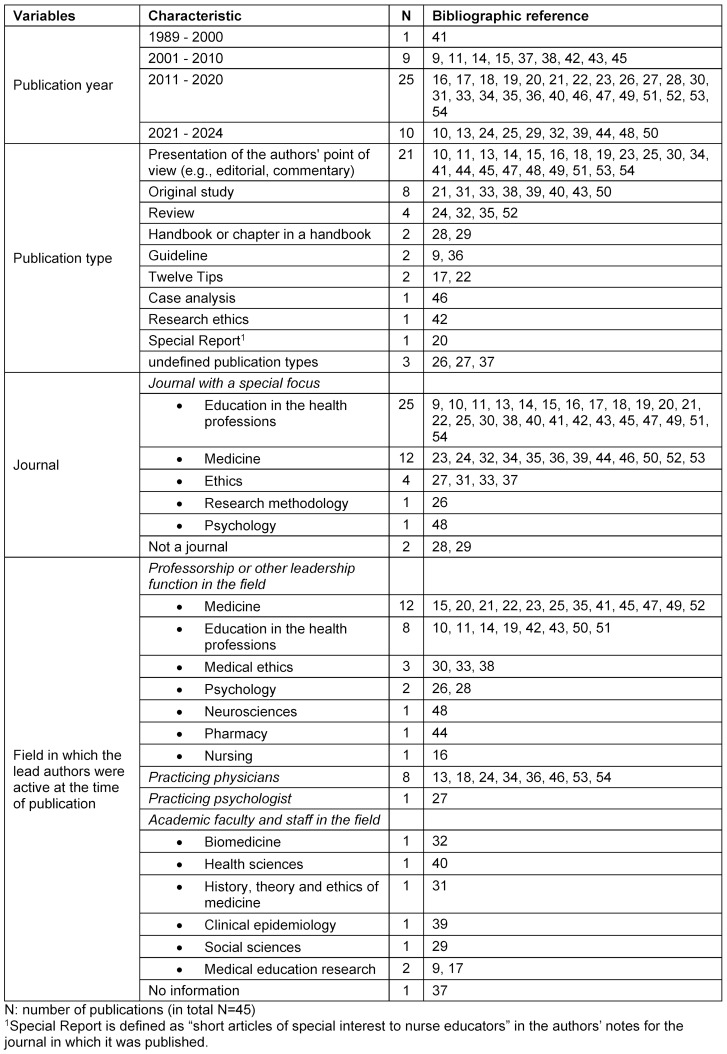
Description of the publications used to compile the problematic areas
