# Supplementary material for: Developing recommendations on ethical aspects affecting studies in health professions education research
Source: GMS J Med Educ. 2026 Jan 15;43(1):Doc1. doi: 10.3205/zma001795 (PMC12875059; doi:10.3205/zma001795)
Supplement: Supplementary material [file JME-43-1-s-001.pdf]

Attachment 1: Supplementary material

Table A1: Examples of queries for the explorative literature review

Search location	Date of search	Search terms
**Database**		
PubMed	24/11/2023	("medical education research"[TIAB] OR "medizinische Ausbildungsforschung"[TIAB] OR "health professional education research" [TIAB]) AND ("research ethics"[TIAB] OR "ethical standards"[TIAB] OR "Ethik"[TIAB] OR "ethical conduct"[TIAB] OR "standards of ethics"[TIAB] OR "code of conduct"[TIAB] OR "Ethics, Research"[MH] OR "ethics"[TIAB] OR "Codes of Ethics"[MH])
ERIC	24/11/2023	"education Research" AND medical AND ethic*
Fachportal Pädagogik	24/11/2023	(Medical Education OR Medizinische Ausbildung) AND (Research OR Forschung) AND (Ethik OR ethics)
**Journal archive**		
Journal of Medical Ethics	24/11/2023	"Medical Education" and in a separate query "Medical Education Research"
**Search engine**		
Google Scholar	07/12/2023	"interview expert* Forschungsethik"
Google	07/12/2023	Each as separate queries: "Befragung Ethik medizinische Ausbildungsforschung"; "Ethik medizinische Ausbildungsforschung Experteninterview"; "Ethik medizinische Ausbildungsforschung Experteninterview"

Figure A2: Flowchart of the process for discussing and deciding on the problematic areas in the online working meetings of the Ethics WG

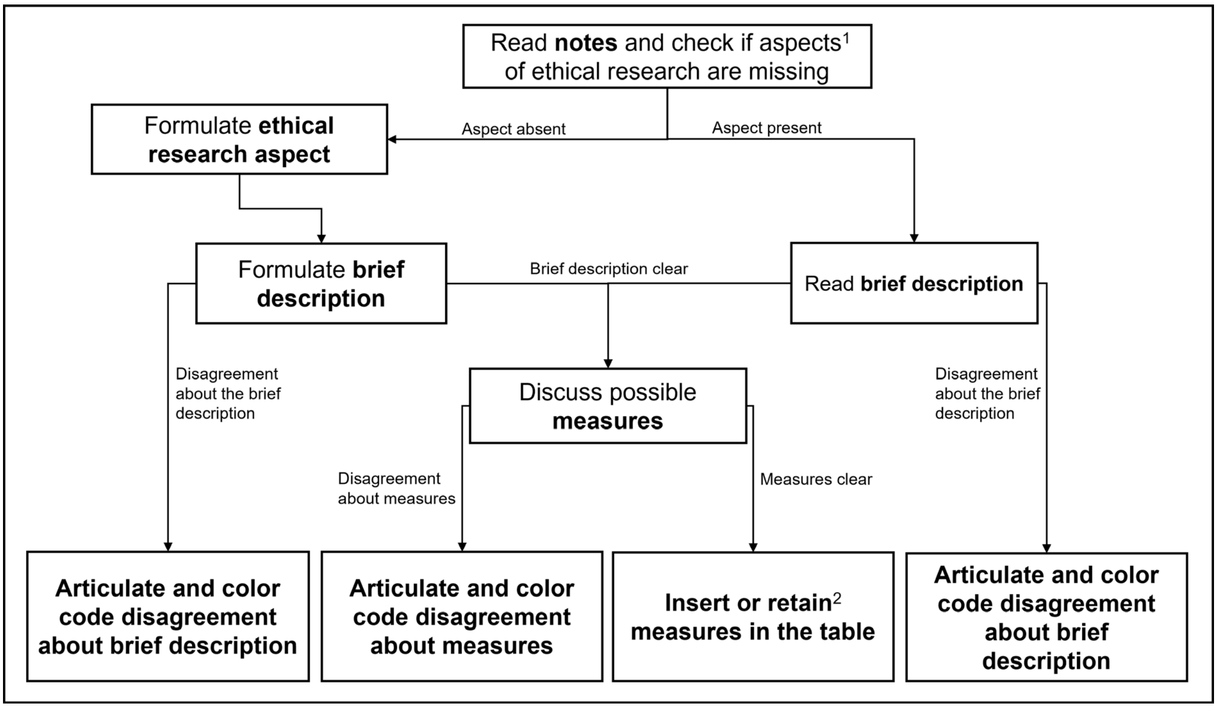

Notes: [1]The problematic areas break down into aspect of ethical research which are each explained in the brief description. The measures for an aspect specify what researchers can do to follow the principles of ethical research. [2] The word "retain" was added after the first two online working meetings because the problematic areas and proposed measures were already being worked on in the subsequent meetings.

Figure A3: Criteria for expert selection

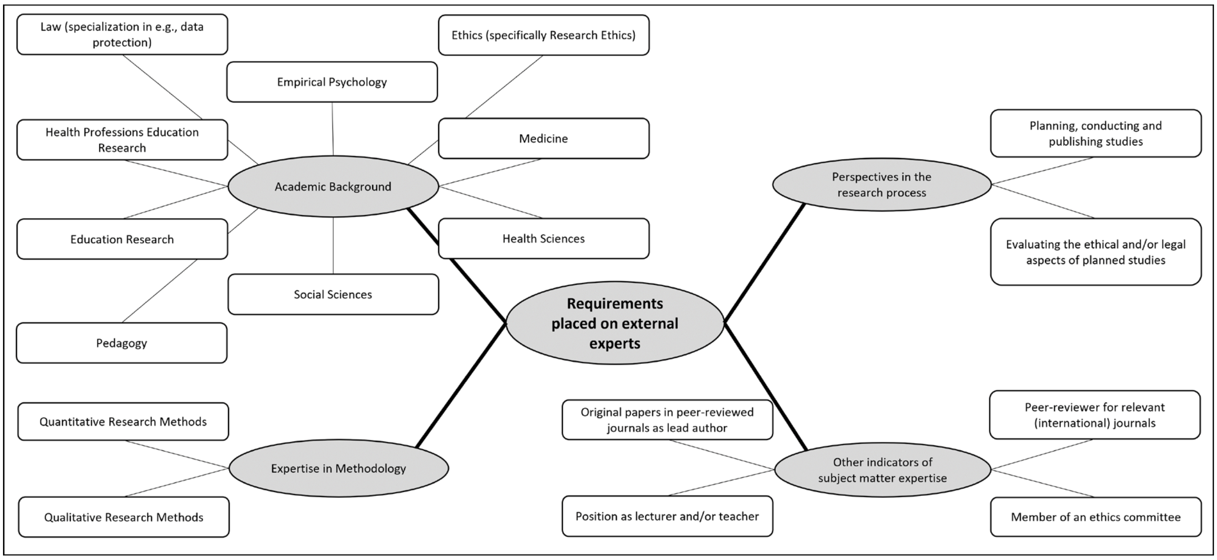

Note: Experts should match at least one sub-item for at least two of the four areas.

Figure A4: Flowchart showing the explorative search for and selection of literature at the beginning of the process

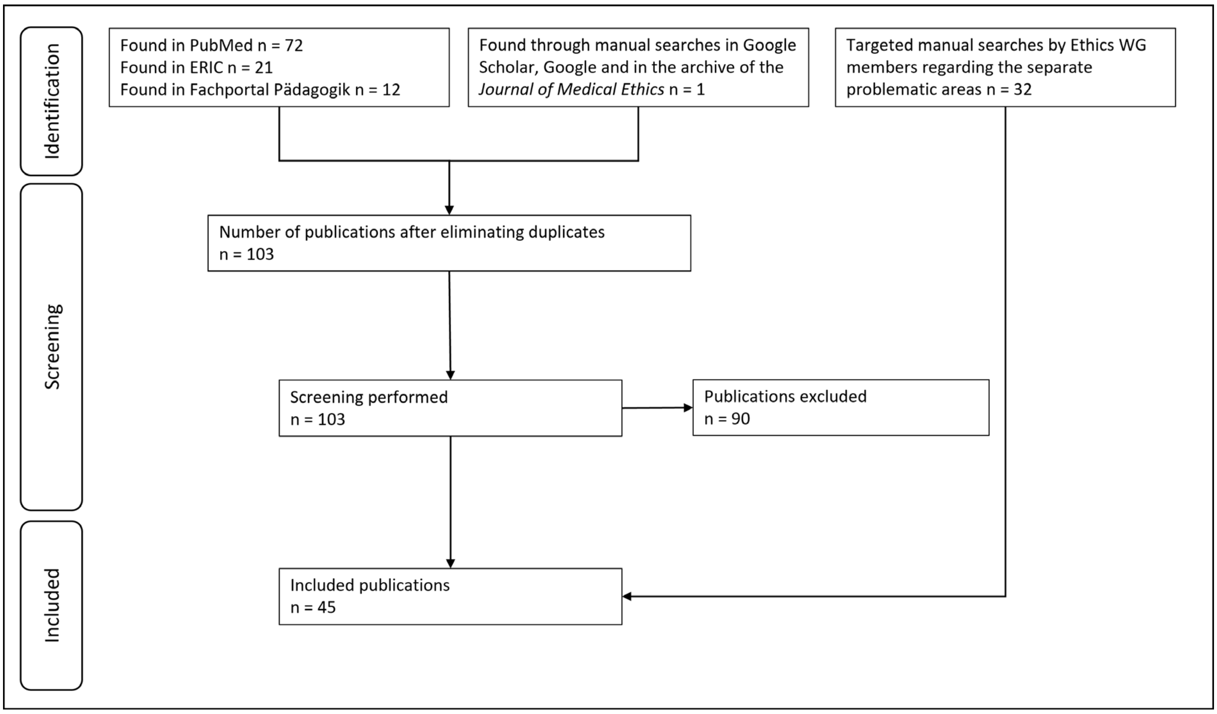

Attachment 1 to Hirsch J, Giesler M, Matthes J, Homberg A, Himmelbauer M, Bauer D, Boeker M, Schüttpelz-Brauns K. *Developing recommendations on ethical aspects affecting studies in health professions education research*. GMS J Med Educ. 2026;43(1):Doc1. DOI: 10.3205/zma001795
