# Supplementary material for: Developing recommendations on ethical aspects affecting studies in health professions education research
Source: GMS J Med Educ. 2026 Jan 15;43(1):Doc1. doi: 10.3205/zma001795 (PMC12875059; doi:10.3205/zma001795)
Supplement: Problematic areas [file JME-43-1-s-002.pdf]

Attachment 2: Problematic areas

Problematic area 1: Research methodology

Background information

Different research methods and study designs are used in health professions education research. The methods predominantly come from the fields of social science and education science. Each study design and each method poses its own ethical challenges. To recognize these challenges and handle them properly, scientists need to have expertise and experience with the intended study design and methods.

Ethical research aspects in this area

Research question(s)

Brief description: The research question(s) is/are at the heart of the study and is/are strongly indicative of the methods for data collection and analysis. The formulation of a relevant research question takes time and requires thorough groundwork in the form of a literature review. A research question is relevant when there are no findings, or only few or outdated findings concerning the research topic in the current literature and when it can be shown, based on the literature, that answering the research question will contribute to the body of knowledge in education research and/or improve education in the health professions.

Researchers' methodological competence

Brief description: In order to recognize the ethical aspects of a study design and the research method(s) and handle them properly, it is necessary to have accurate and detailed knowledge and basic experience with the design and methods. Furthermore, reliable results can only be obtained if the researchers have the commensurate methodological competence.

Bias

Brief description: The aim of health professions education research is to produce reliable and trustworthy results that contribute to high-quality education and/or further develop and advance education. Researchers should therefore take precautions to keep any bias as slight as possible.

Study protocol

Brief description: The study protocol contains a detailed study plan. It serves not only to reduce bias, but also to improve the reproducibility and consistency of the methods. Changes to this document must be recorded and justified. Publishing the study protocol increases the certainty that the researchers will follow the planned procedure. Thus, it is not only clear how the results were arrived at, but the researchers are also required to justify any modifications to or deviations from the study procedure and/or methods. This encourages adherence to ethical research practices.

Literature

Artino ARJ, Driessen EW, Maggio LA. Ethical Shades of Gray: International Frequency of Scientific Misconduct and Questionable Research Practices in Health Professions Education. Acad Med. 2019; 94(1):76-84. DOI: 10.1097/ACM.0000000000002412

Bergold J, Thomas S. Partizipative Forschungsmethoden: Ein methodischer Ansatz in Bewegung. Forum Qualitative Sozialforschung. 2012; 13(1). DOI: 10.17169/fqs-13.1.1801

DuBois JM, Antes AL. Five Dimensions of Research Ethics: A Stakeholder Framework for Creating a Climate of Research Integrity. Acad. Med. 2018; 93 (4): 550-555. DOI: 10.1097/ACM.0000000000001966

Hally E, Walsh K. Research ethics and medical education. Med Teach. 2016;38(1):105-6. DOI: 10.3109/0142159X.2014.956068

Panter AT, Sterba SK. Handbook of Ethics in Qauntitative Methodology. New York:Routledge; 2011.

Schüttpelz-Brauns K, Koch R, Mertens A, Stieg M, Boonen A, Marienhagen J. Ethik in der Medizinischen Ausbildungsforschung. GMS Z Med Ausbild. 2009;26(1):Doc08. DOI: 10.3205/zma000600

Ten Cate O. The Ethics of Health Professions Education Research: Protecting the Integrity of Science, Research Subjects, and Authorship. Acad Med. 2022; 97(1):13-17. DOI: 10.1097/ACM.0000000000004413.

Tzanetakis M. Qualitative Online-Forschungsmethoden: Digitale Datenerhebung und ethische Herausforderungen. In: Prainsack B, Pot M, Hrsg. Qualitative und interpretative Methoden in der Politikwissenschaft. Wien: Facultas; 2021. p. 130-141.

Wasserman R. Ethical Issues and Guidelines for Conducting Data Analysis in Psychological Research. Ethics Behav. 2013; 23(1), 3–15. DOI: 10.1080/10508422.2012.728472

Problematic area 2: Compliance with laws and guidelines

<u>Background information</u>
Guidelines, laws and regulations are the operationalization of ethical and moral principles. Researchers must follow locally valid laws, guidelines and codes of practice. Additional standards may be relevant in the case of joint projects. A basic tenet of research is that harm should be avoided to the greatest extent possible and that risks should only be taken if the potential benefits to the individual and society outweigh them. Education research, however, poses its own risks.

<u>Ethical research aspects in this area</u>

Lack of knowledge about regional laws and regulations
Brief description: It is possible that researchers are not sufficiently informed about the specific legal requirements that apply in their region or to their study.

Insufficient information given to the participants
Brief description: It is possible that participants are not informed in enough detail about the study, its procedure or the potential advantages and disadvantages.

Data collection and pseudonymization
Brief description: The gathering of non-anonymous data, such as images, videos or audio recordings, and exact transcriptions (including any potential conclusions based on specific language or slang), can reveal the identities of the participants, even when pseudonyms are used.

Secondary data and consent
Brief description: The challenge when using secondary data is to ensure that consent has been obtained from the affected individuals and that ethical standards are followed.

Insecure online surveys and data storage locations
Brief description: The use of insecure online surveys or untrustworthy data storage locations can pose risks to the participants' security and privacy.

Handling of social media in research
Brief description: Lack of clarity on the rules for handling social media in research projects can lead to ethical issues, especially in regard to data protection and participant consent.

Transparency in research
Brief description: The failure to include responsible individuals (e.g., the staff council in the case of employee surveys; the quality management team or institutional management in the case of student evaluations).

Literature

Egan-Lee E, Freitag S, Leblanc V, Baker L, Reeves S. Twelve tips for ethical approval for research in health professions education. Med Teach. 2011; 33(4):268-72. DOI: 10.3109/0142159X.2010.507715

Ferrero F. Some ethical aspects of medical education research. Arch Argent Pediatr. 2018; 116(6):382-5. DOI: 10.5546/aap.2018.eng.384

Guckian J, Thampy H. Gaining ethics approval for health professions education research. Clin Exp Dermatol. 2022; 47(12):2081-4. DOI: 10.1111/ced.15185

Klitzman RL. Understanding Ethical Challenges in Medical Education Research. Acad Med. 2022; 97(1):18-21. DOI: 10.1097/ACM.0000000000004253

Problematic area 3: Good scientific practice

Background information
As in other scientific disciplines, it is also important for health professions education researchers to participate in the academic discourse and publish their research findings. To begin with, the target audience needs to be informed about the research results; and furthermore, the current body of knowledge on the various topics is meant to be expanded and augmented. The publication of study findings contributes not only to an improvement in education in the health professions, but also to the further development of the field of education research. Ethical aspects must also be taken into account when publishing.

Ethical research aspects in this area

Authorship
Brief description: Authors are the individuals who have made a real and visible contribution to the content of a publication, e.g., by collaborating on the development of the research project, gathering or analyzing data and/or writing the paper. This rules out honorary authorship, co-authorship on the basis of leadership function, and including an author because of their prominence in a field of research. If the individuals participating in the research have conflicts of interests, these must be disclosed in the publication (see conflicts of interest); ghostwriting is thus not an option.

Writing the paper
Brief description: As mentioned in the background information for this problematic area, there are various recommendations that provide information on the structure and content for the intended publication. Generally, both the methods and the results must be presented without alterations so that others can arrive at an overall and, above all, undistorted understanding of the research subject matter.

Citation
Brief description: As part of a study, health professions education researchers delve into the literature on a research topic. Relevant content in other papers is included in the publication of their own study results so as to embed their research in the scientific discourse, document their line of argumentation and make their views clear.

Conflicts of interest
Brief description: Conflicts of interest on the part of health professions education researchers must be disclosed so that the results of a study can be assessed appropriately. An education researcher's primary interest should be to objectively investigate the research subject matter and report the results in an unbiased manner. However, it is possible that secondary interests influence the research activities. Possible secondary interests include: economic or personal connections to businesses or institutions for whom the study is relevant, as well as political, academic or personal interests that concern the paper.

Submitting a paper for publication
Brief description: Attention must also be paid to the integrity and transparency of the research when submitting a paper to a professional journal.

Availability of research data
Brief description: Comprehensibility and reproducibility are meant to be increased by making research data (publicly) accessible. The range of the research data can also be increased. The FAIR Guiding Principles (Findable, Accessible, Interoperable, Reusable) should be followed when making research data available.

Literature

Artino ARJ, Driessen EW, Maggio LA. Ethical Shades of Gray: International Frequency of Scientific Misconduct and Questionable Research Practices in Health Professions Education. Acad Med. 2019; 94(1):76-84. DOI: 10.1097/ACM.0000000000002412

Bion J, Antonelli M, Blanch L, Curtis JR, Druml C, Du B, Machado FR, Gomersall C, Hartog C, Levy M, Myburgh J, Rubenfeld G, Sprung C. White paper: statement on conflicts of interest. Intensive Care Med. 2018; 44(10):1657-1668. DOI: 10.1007/s00134-018-5349-8.

Caelleigh AS. Roles for scientific societies in promoting integrity in publication ethics. Sci Eng Ethics. 2003; 9(2):221-41. DOI: 10.1007/s11948-003-0010-4.

Fuerholzer K, Schochow M, Peter R, Steger F. Medical Students' Acquaintance with Core Concepts, Institutions and Guidelines on Good Scientific Practice: A Pre- and Post-questionnaire Survey. Sci Eng Ethics. 2020; 26: 1827–1845. DOI: 10.1007/s11948-020-00215-3

Heitman E, Olsen CH, Anestidou L, Bulger RE. New graduate students' baseline knowledge of the responsible conduct of research. Acad Med. 2007; 82(9):838-45. DOI: 10.1097/ACM.0b013e31812f7956.

Hofmann B, Helgesson G, Juth N, Holm S. Scientific Dishonesty: A Survey of Doctoral Students at the Major Medical Faculties in Sweden and Norway. J Empir Res Hum Res Ethics. 2015; 10(4):380-8. DOI: 10.1177/1556264615599686

Johal J, Loukas M, Oskouian RJ, Tubbs RS. "Political co-authorships" in medical science journals. Clin Anat. 2017; 30(6):831-834. DOI: 10.1002/ca.22932.

Kahn NB, Lichter AS. The new CMSS code for interactions with companies managing relationships to minimize conflicts. J Vasc Surg. 2011; 54(3):34S-40S. DOI: 10.1016/j.jvs.2011.05.109.

Kearney M, Downing M, Gignac EA. Research integrity and academic medicine: the pressure to publish and research misconduct. J Osteopath Med. 2024; 124(5):187-194. DOI: 10.1515/jom-2023-0211.

Mubeen SM, Qurrat-ul-Ain, Ghayas R, Adil Rizvi SH, Khan SA. Knowledge of scientific misconduct in publication among medical students. Educ Health (Abingdon). 2017; 30(2):140-145. DOI: 10.4103/efh.EfH_221_16.

Nishimura R, Takeuchi J, Sakuma M, Uchida K, Higaonna M, Kinjo N, Sakakibara F, Nakamura T, Kosaka S, Yoshimura S, Ueda S, Morimoto T. Experience and awareness of research integrity among Japanese physicians: a nationwide cross-sectional study. BMJ Open. 2021; 11(10):e052351. DOI: 10.1136/bmjopen-2021-052351.

Petersdorf RG. A matter of integrity. Acad Med. 1989; 64(3):119-23. DOI: 10.1097/00001888-198903000-00003.

Problematic area 4: Competence of ethics committees

Background information

Ethical review by an ethics committee is necessary when, e.g., several institutions collaborate on a study, an experimental study design in involved, generalizable results are aimed for and the results are intended for publication. The assessment of an ethics committee is desirable if an evaluation is involved that contributes to improving teaching or research on/with people in a normal educational setting and anonymized data is collected. Most professional journals usually require proof of ethical approval for all studies on humans. If no ethical review is possible, then it is common to present complete and exact information on how participants were treated.

Ethical research aspects in this area

Lack of rules and guidelines for approving studies in health professions education research

Brief description: The responsibility for conducting ethically correct research lies with the researchers. The responsibility for deciding which papers get published lies with the responsible individuals at a professional journal. The ethics committee needs to have expertise in education research.

Aptitude of local ethics committees

Brief description: The suitability of local (medical) ethics committees to review and approve studies in health professions education research is unclear. However, an independent review of education research studies in the health professions is needed. The approving body should possess subject matter competence in education research.

Refusal to review by the competent ethics committee

Brief description: For various reasons it is possible that a review cannot be undertaken by the competent ethics committee or that it declines to conduct a review. This can pose an obstacle to the course of the study and/or publication.

Literature

Albon SP, Hu F. Guiding Ethics Review in Pharmacy Education Research and Scholarship at UBC: Clarifying the Unclear. Innov Pharm. 2021; 12(3). DOI: 10.24926/iip.v12i3.3919

Boileau E, Patenaude J, St-Onge C. Twelve tips to avoid ethical pitfalls when recruiting students as subjects in medical education research. Med Teach. 2018; 40(1):20-5. DOI: 10.1080/0142159X.2017.1357805

Brown J, Ryland I, Howard J, Shaw N. Views of National Health Service (NHS) Ethics Committee members on how education research should be reviewed. Med Teach. 2007; 29(2-3): 225-230. DOI: 10.1080/01421590701300179

Chen RP. Student participation in health professions education research: in pursuit of the Aristotelian mean. Adv Health Sci Educ Theory Pract. 2011; 16(2):277-86. DOI: 10.1007/s10459-009-9164-4

Egan-Lee E, Freitag S, Leblanc V, Baker L, Reeves S. Twelve tips for ethical approval for research in health professions education. Med Teach. 2011; 33(4):268-72. DOI: 10.3109/0142159X.2010.507715

Eva KW. Research ethics requirements for Medical Education. Med Educ. 2009;43(3):194-5. DOI: 10.1111/j.1365-2923.2008.03285.x

Ferrero F. Some ethical aspects of medical education research. Arch Argent Pediatr. 2018; 116(6):382-5. DOI: 10.5546/aap.2018.eng.384

Hally E, Walsh K. Research ethics and medical education. Med Teach. 2016;38(1):105-6. DOI: 10.3109/0142159X.2014.956068

Kanter SL. Ethical approval for studies involving human participants: academic medicine's new policy. Acad Med. 2009; 84(2):149-50. DOI: 10.1097/ACM.0b013e318198c40f

McLachlan J, McHarg J. Ethical permission for the publication of routinely collected data. Med Teach. 2005; 39(9): 944–948. DOI: 10.1111/j.1365-2929.2005.02223.x

Morrison J, Prideaux D. Ethics approval for research in medical education. Med Educ. 2008; 35(11): 1008. DOI: 10.1046/j.1365-2923.2001.01076.x

Schutte T, Scheele F, van Luijk S. Roses and Balances: A Paradigm for Constructive Ethical Review of Health Professions Education Research. Adv Med Educ Pract. 2021; 12: 529-535. DOI: 10.2147/AMEP.S305094

Ten Cate O. Why the ethics of medical education research differs from that of medical research. Med Educ. 2009; 43(7): 608-610. DOI: 10.1111/j.1365-2923.2009.03385.x

Problematic area 5: Students as a vulnerable group

<u>Background information</u>
Students and trainees in the health professions may exhibit certain characteristics that make them vulnerable and in need of protection under research conditions. It is important to consider the research context, the type of study and the individual circumstances of the potential participants and study participants.

<u>Ethical research aspects in this area</u>

Psychological stress and health
Brief description: Students and trainees can be more susceptible to stress and mental health issues compared to the general public because they are often under pressure to perform.

Pressure to participate and coercion
Brief description: Peers and the surrounding environment can exert strong pressures to participate in studies or research activities, especially if these take place in connection with required courses.

Power imbalances and hierarchies
Brief description: The existing power imbalance between teachers/researchers and students/trainees can lead to an unequal relationship in which the students/trainees may not feel free to express their opinions or to refuse to participate in a study.

Possible disadvantages of participation
Brief description: Participation in studies can have negative consequences, for instance, success in learning and on exams can decrease, time can be lost, study participation can cause emotional stress due to the treatment of sensitive topics or have physical effects as a result of examinations performed on one's own body.

Lack of compensation and confidentiality
Brief description: A lack of compensation for potential disadvantages, particularly in the case of studies that take place during the semester and involve required courses, as well as a lack of confidentiality about participation can put additional stress and pressure on students.

Risk of stigmatization
Brief description: Individuals and groups of people may be stigmatized by the collection and analysis of sensitive data. There is the risk of discrimination. The risks and benefits need to be weighed against each other prior to publication.

Need for fairness, diversity and integration
Brief description: It is necessary to ensure fairness, diversity and inclusion when recruiting participants, and collecting and analyzing data.

Incentives for study participation
Brief description: Reward/incentives for study participation can increase the pressure on individuals who are interested and those who are not interested in participating in the study.

Scheduling the data collection
Brief description: The time point of data collection, especially if it is just before important assessments, can cause additional stress and lower willingness to participate.

Literature

Cutting LE. Contextualizing school achievement among vulnerable learners: Implications for science and practice. New Dir Child Adolesc Dev. 2022; 2022(183-184): 91-94. DOI: 10.1002/cad.20460

Dolan BM, Arnold J, Green MM. Establishing Trust When Assessing Learners: Barriers and Opportunities. Acad Med. 2019; 94(12): 1851-1853. DOI: 10.1097/ACM.0000000000002982

Ferrero F. Some ethical aspects of medical education research. Arch Argent Pediatr. 2018; 116(6):382-5. DOI: 10.5546/aap.2018.eng.384

Guckian J, Thampy H. Gaining ethics approval for health professions education research. Clin Exp Dermatol. 2022; 47(12):2081-4. DOI: 10.1111/ced.15185

Hauer KE, Lucey CR. Core Clerkship Grading: The Illusion of Objectivity. Acad Med. 2019; 94(4): 469-472. DOI: 10.1097/ACM.0000000000002413

Heflin MT, DeMeo S, Nagler A, Hockenberry MJ. Health Professions Education Research and the Institutional Review Board. Nurse Educ. 2016; 41(2):55-9. DOI: 10.1097/NNE.0000000000000230

Klitzman RL. Understanding Ethical Challenges in Medical Education Research. Acad Med. 2022; 97(1):18-21. DOI: 10.1097/ACM.0000000000004253

Torralba KD, Jose D, Byrne J. Psychological safety, the hidden curriculum, and ambiguity in medicine. Clin Rheumatol. 2020; 39(3):667–671. DOI: 10.1007/s10067-019-04889-4

Problematic area 6: Dealing with other study populations

Background information

Not only are data on students/trainees in the health professions important for health professions education research, but also data from other study populations. When dealing with other study populations, challenges and barriers may arise and which researchers will have to manage.

Ethical research aspects in this area

Patients

Brief description: Patients may be study participants or indirectly affected by the study. In both cases, researchers face a wide variety of challenges to ensure safety, privacy and data protection.

Teachers

Brief description: Teachers may be study participants or indirectly affected by the study. In both cases, researchers face a wide variety of challenges to ensure safety, privacy and data protection.

Members of interprofessional teams

Brief description: Interprofessional team members may be study participants or indirectly affected by the study. In both cases, researchers face a wide variety of challenges to ensure safety, privacy and data protection.

Assistant physicians

Brief description: Assistant physicians are undergoing specialist training and can/should also be part of education research studies. The employment situation and workload, as well as other factors, should be taken into account in the planning and conduction of the study when including this group.

Minors

Brief description: Minors may be study participants or indirectly affected by the study. In both cases, researchers face a wide variety of challenges to ensure safety, privacy and data protection.

Pregnant women

Brief description: Pregnant women may be study participants or indirectly affected by the study. In both cases, researchers face a wide variety of challenges to ensure, above all, safety, privacy and data protection.

Literature

Keune JD, Brunsvold ME, Hohmann E, Korndorffer JR Jr, Weinstein DF, Smink DS. The ethics of conducting graduate medical education research on residents. Acad Med. 2013; 88(4):449-53. DOI: 10.1097/ACM.0b013e3182854bef

Klitzman RL. Understanding Ethical Challenges in Medical Education Research. Acad Med. 2022; 97(1):18-21. DOI: 10.1097/ACM.0000000000004253

Kraus CK, Guth T, Richardson D, Kane B, Marco CA. Ethical considerations in education research in emergency medicine. Acad Emerg Med. 2012; 19(12):1328-32. DOI: 10.1111/acem.12019

Kumar VD, Murugan M. Professionalism in practice: Exploring the ethical perplexity of involving students in Medical Education Research. J Adv Med Educ Prof. 2020; 8(4):200-203. DOI: 10.30476/jamp.2020.74921.0

Leentjens AF, Levenson JL. Ethical issues concerning the recruitment of university students as research subjects. J Psychosom Res. 2013; 75(4):394-8. DOI: 10.1016/j.jpsychores.2013.03.007.

Moreau KA, Eady K, Heath SE. Patient Involvement in Medical Education Research: Results From an International Survey of Medical Education Researchers. J Patient Exp. 2021; 8. DOI: 10.1177/2374373520981484

Schutte T, Scheele F, van Luijk S. Roses and Balances: A Paradigm for Constructive Ethical Review of Health Professions Education Research. Adv Med Educ Pract. 2021; 12: 529-535. DOI: 10.2147/AMEP.S305094

Walsh K. Medical education research: is participation fair? Perspect Med Educ. 2014;3(5):379-82. DOI: 10.1007/s40037-014-0120-5
