# Supplementary material for: Developing recommendations on ethical aspects affecting studies in health professions education research
Source: GMS J Med Educ. 2026 Jan 15;43(1):Doc1. doi: 10.3205/zma001795 (PMC12875059; doi:10.3205/zma001795)
Supplement: Recommendations on ensuring data protection, free consent, no harm to and autonomy of par-ticipants and potentially affected individuals (rec-protect) [file JME-43-1-s-003.pdf]

Attachment 3: Recommendations on ensuring data protection, free consent, no harm to and autonomy of participants and potentially affected individuals (rec-protect)

Table: Legend for the research process icons

Icon	Stage in the research process
	Survey of topics & state of research
	Identification of a research gap
	Formulation of a research question
	Study planning
	Recruitment
	Data collection
	Data analysis
	Writing the paper
	Measure affects the entire research process

Explanations

- Each recommended measure has been assigned an identifier. The logic behind these identifiers is as follows:
  Short version of title_area number.measure number
- Icons have been created to give a clear overview of the time points at which each measure is relevant in the research process.

Note

The recommendations should be understood to supplement existing guidelines and codes of conduct. They are based on the local perspectives of the authors (DE, CH, AT) are aimed toward the GDPR and other regulations. Users should check the rules and regulations applying to them and adapt these recommended measures accordingly.

Attachment 3 to Hirsch J, Giesler M, Matthes J, Homberg A, Himmelbauer M, Bauer D, Boeker M, Schüttpelz-Brauns K. *Developing recommendations on ethical aspects affecting studies in health professions education research.* GMS J Med Educ. 2026;43(1):Doc1. DOI: 10.3205/zma001795

1) Observing Data Minimization in regard to Personal Data	
Rec-Protect_1.1<br><br>The research team should determine if collecting personal data and/or sensitive personal data is necessary to answer the research question and/or describe the sample.	**Definitions:**<br>• *Data subject:* Natural persons whose personal data are processed (in the context here of a research question).<br>• *Personal data* is information that enable the direct or indirect identification of a data subject, such as name, birthdate, address and other contact information, or physical characteristics (Art. 4 (1) GDPR).<br>• *Sensitive personal data* (also referred to as "special categories of personal data" in Art. 9 (1) GDPR) is information which not only enables definitive identification of a person, but also can entail data particularly worthy of protection, such as ethnic origin and information concerning health or religious beliefs. This information is considered especially worthy of protection because its divulgence can pose substantial risks to the data subject.<br>• *Data minimization:* The amount of personal information gathered for processing must kept to the minimum necessary to fulfill the purpose (i.e. answering the research question) (Art. 5 (1)(c) GDPR).<br>**Reason:** Determining whether and to what extent personal data and/or sensitive personal data are necessary to answer the research question and/or describe the sample is the prerequisite for proper planning and conduction of the study.<br>**Notes:**<br>• In quantitative data collection and statistical analysis, the composition of the sample is described to verify if it is representative of the population. Sociodemographic data is gathered on the participants for this reason. It is important here to carefully check if collecting and processing this data can be done in a pseudonymized or anonymized manner. If there is no anonymity, then data protection law must be followed.<br>• If the research question requires qualitative research methods to collect and analyze data, this usually involves gathering personal data and therefore necessitates confidentiality and compliance with data protection regulations when planning, conducting, and publishing the study.<br>• Whether or not researchers apply quantitative or qualitative research methods, the mode of data collection is crucial to ascertain if the data collection involves personal or anonymized data. Images, videos and audio recordings, for example, are always personal data making it necessary to comply with data protection law when processing them. This also applies when these data are anonymized later in the study.
Rec-Protect_1.2<br><br>The research team must ensure that the processing of personal data has an adequate legal basis.<br><br>If data processing is based on consent, the data subject must be provided the information stipulated by the GDPR about the data processing.	**Definition:** The processing of personal data must be undertaken legally, whereby Art. 6 GDPR lists the conditions under which legally compliant processing can occur. Art. 9 GDPR must also be adhered to in the case of particularly sensitive data. In most cases, data processing will be based on consent by the data subject; however, in individual cases it can also take place on a legal basis or based on legitimate interest.<br>If personal data processing is based on the consent of the data subject, the information listed in Art. 13 GDPR must be given to the person in advance.<br>**Reason:** It is mandatory that data processing have a legal basis. If this is not the case, it may result in the need to delete the affected research data or lead to other consequences arising from a violation of the GDPR.<br>**Notes**:<br>• Which legal basis applies depends on the research question and the type of data to be processed. Another criterion can be if the data are already present at the institution. Consulting with the official data protection officer is recommended in individual cases, especially if processing is not be based on consent.<br>• If the data will not be collected anonymously from the data subject, then consent should be obtained in writing (i.e. through personal signature).

Rec-Protect_1.3<br><br>The research project should be carried out with pseudonymized data if, for the purpose of traceability or proving data accuracy, it must be possible to draw conclusions about the data subject.<br><br>If study participants are given pseudonyms, the list assigning the personal data to the pseudonyms should be kept separate from the research data (preferably with a trusted third party, as is possible at most university hospitals) and thus protected so that only authorized individuals have access to the respective data.	**Definition:** A *pseudonym* is a fictive designation (normally a non-semantic identifier) for a person that is used to remove direct and indirect identifying attributes from the data set (i.e. conceal the identity of participants), yet still retain the connections between the data and the study participants. The personal data are saved in lists together with the pseudonyms. Ideally, these should be kept by a trusted third party, as is the case at most university hospitals, so that the identifying data are guarded to the extent possible against unauthorized access. The use of pseudonyms makes it possible, e.g., in longitudinal studies, to contact study participants for data collection at different time points and add this data into the existing data sets. Pseudonymization is also a means to guarantee secure, triple-blinding of the researchers involved in the study (e.g., for analyses). Furthermore, pseudonymization also makes it possible to link data from different sources (record linkage), e.g., to combine data from apps and data from questionnaires via survey portals, while at the same time protecting personal privacy, meaning without transmitting identifying characteristics. It is only through pseudonyms that data collected longitudinally at multiple time points can be assigned meaningfully to one person. Currently, there are very secure pseudonymization procedures that technically can and should be used in a centralized manner by the trusted third party.<br>When processing pseudonymized data, it must be noted that these data continue to be considered personal data and are subject to data protection law (Recital 26 GDPR).<br>**Reason:** This measure helps ensure that unauthorized third parties are not given access to the personal data and that the data for a pseudonym cannot be readily reassigned to the study participant. Thus, the identities of the study participants also remain as protected and blinded as possible inside the research team.<br>**Notes:**<br>• Pseudonyms should be used if anonymous data collection and processing or anonymization are not possible.<br>• Pseudonymized data collection and processing is currently the common standard or first choice because it offers the highest possible protection of personal privacy and the maximum range of options for storing and processing the data.<br>• To publish data sets, an anonymized data set can be created through the removal of specific data and other measures (usually reducing the attributes).<br>• The *Guidelines 01/2025 on Pseudonymisation* of the *European Data Protection Board* should be heeded as a basis for planning and carrying out pseudonymization.
Rec-Protect_1.4<br><br>If the research question can be answered using anonymous data, the research team should ensure that<br>• only anonymous data is collected with the data collection instrument, and<br>• the identities of the study participants are not revealed by transmitting the data to the research team.	**Definition:** *Anonymous data*, according to the GDPR, is identifying personal data that are gathered and processed in such a way that makes it impossible to identify the data subject or possible to do so only through an unreasonable amount of effort and expense (Recital 26 GDPR).<br>**Reason:** Anonymization helps to protect the identities of study participants and facilitate responsible handling of the research data.<br>**Notes:**<br>• There is also a risk of re-identification when research data are gathered without directly identifiable personal data. For example, there could be response options that when selected (even in combination with other information) could enable conclusions to be drawn about specific individuals. Attention must be paid to this aspect when drafting a survey. It is preferable to design the survey so that no conclusions can be drawn. If, due to the line of scientific questioning, detailed information is desired that carries the risk of identification and if this data is collected from the data subject, then information explaining this risk should be provided.<br>• For online surveys, this measure includes ensuring that the tool used does not record any personal data, such as the IP address.<br>• If personal data becomes visible during data transmission, e.g., as a result of returning research data via email to the research team, then the participants' anonymity no longer exists.<br>• If the questionnaire contains free-text boxes, the participants should be told not to share any personal information in them. It should be checked to see if having this option in the questionnaire requires additional data protection measures.<br>• In the informational letter about the study, it should be pointed out that data which has been collected anonymously cannot be deleted afterwards at the request of the participants.<br>• Even when anonymous research data is intended for publication, e.g., in a repository, after study completion this data should be stored locally to ensure data quality (as a measure against intentional and unintentional modification to or manipulation of the data). This demonstrates responsible handling of the collected data.<br>• An increased risk of re-identification is posed by de-identified data sets with many attributes, texts, images or videos (in which direct and indirect personally attributable characteristics have been deleted or anonymized). Whether the anonymization fulfills the legal requirements depends on the use case and type

	of data, and is a complex concept for which there is no simple, layman's explanation. As previously mentioned, it is recommended that in cases of uncertainty the study participants be informed of the residual risk of re-identification.<br>• If codes are used to make longitudinal links between data sets without keeping a list to connect the personal data, as is done for pseudonymization (see Rec-Protection_1.3), attention must be paid so that the code does not contain any identifying information (e.g., name, birthdate, matriculation number).

2) Protecting Study Participants' Personal Data and Sensitive Personal Data	
Rec-Protect_2.1<br><br>The research team should be familiar with the institution's internal rules concerning data protection. When personal data is processed, the official data protection officer should be involved at an early stage.	**Definition:** The official data protection officer is actively involved within an institution in the compliance with data protection law and monitors the processes for protecting data. The institution's management is usually responsible for data protection. The data protection officer advises the management in regard to compliance with data protection law. The data protection officer is not a member of the research team.<br>**Reason:** This measure helps ensure that the research team knows and follows the institution's internal rules for data protection and is in contact with the official data protection officer.<br>**Notes:**<br>• If the research team is uncertain about whether or not the data to be collected for the study involve personal data and/or is uncertain about the internal rules for data protection and/or about the need to include the data protection officer, these questions should be raised and clarified directly with the data protection officer and/or the responsible ethics committee.<br>• The ethics committee can insist on the inclusion of the data protection officer. In general, an ethics committee will follow the vote of the data protection officer and, e.g., not recommend the conduction of studies if there are issues concerning compliance with data protection law.
Rec-Protect_2.2<br><br>If personal data and/or sensitive personal data are collected to answer the research question, a detailed concept for protecting this data can be developed, in coordination with the data protection officer, based on the data protection laws applicable to the research team. This concept can outline the procedures for gathering, storing and processing these data.<br><br>If a decision is made not to develop an overall concept for protecting data, then the most important aspects of data processing should be laid down in the study protocol, e.g., legal basis for the data processing, the responsible individuals, data minimization measures (anonymization or pseudonymization). The requirements for documentation imposed by the ethics committee should also be followed in regard to this.	**Definition:** A data protection concept describes, in particular, the measures taken to protect personal data in the context of a research project and ensures compliance with legal obligations (such as protecting the rights of data subjects).<br>**Content of a data protection concept:**<br>• Legal basis for the data processing (generally the consent of the data subject)<br>• Explanation of how the individuals interested in study participation are informed about the data collection and processing and their rights<br>• Type of data collection and processing and its purpose<br>• Specification of the technical and organizational measures (TOMs) to ensure data security (e.g., transmission of data from the study participants to the research team, authorizations to access the data, measures to anonymize or pseudonymize the data, type, duration and security of data storage). These TOMs are defined in more detail based on the description of how the data are handled and are often of a technical nature (close cooperation with IT may be necessary).<br>• Information about the option to withdraw consent to participate in a study and the address of the office responsible for handling such withdrawals<br>• How to handle personal data which has already been collected when a study participant terminates participation or desires to have their data erased (withdrawal of consent).<br>• Information on the procedure to erase data or restrict data processing when, at the same time, there is an obligation to archive research data.<br>• Regular checks and controls of the implemented data protection measures<br>• Rules for transferring data to third parties; explicit identification of planned transfers to so-called insecure third countries (GDPR) when cooperating with others (e.g., the USA)<br>• References to transparency portals on which research projects must be presented in a transparent manner for the study participants (e.g., trial registers)<br>• Contractual aspects, especially in the case of cooperative ventures with third parties or when using contracted workers (e.g., use of a specific IT tool for surveys)<br>• Data storage and archiving after study completion, as well as data erasure.<br>**Reason:** The development of a data protection concept in accordance with the applicable data protection law helps ensure that all relevant aspects of data protection have already been taken into account when planning the study. In addition, the concept serves as a basis for documentation and proof of the measures undertaken to protect the data.<br>**Notes**:<br>• If a comprehensive concept for data protection should be developed, it is recommended that the appropriate IT colleagues and the official data protection officer are included directly and early on in the process, so that the scope can be defined and obstacles removed. A data protection concept is based on an interdisciplinary process (law, IT, pedagogy and research methodology) and is therefore complex, but often simpler to implement than expected.<br>• Other content can be important for the data protection concept and which is not listed above under "Content of a data protection concept."

Rec-Protect_2.3<br><br>The research team should ensure that all of the people involved in the study have been informed about the data protection laws applicable to the study, the data protection concept based thereon and that they are legally obligated to comply.<br><br>In the case of collaborative research between institutions that entails the processing of personal data, signing a contract designating joint responsibility for the processing is also recommended (Art. 26 GDPR).	**Reason:** This measure helps ensure that the personal data of the study participants is protected in the best way possible and that the applicable provisions of data protection law are observed. Furthermore, it serves to clearly assign each responsibility associated with data protection law.<br>**Notes:**<br>• This measure is recommended not only for the processing of personal and/or sensitive personal data in the context of primary data collection, but also for secondary data analysis.<br>• In addition, the individuals who are bound to professional secrecy (such as medical confidentiality) should *without fail* be informed of its legal validity and intensified rules. In Germany, for example, violations are punishable under criminal law according to § 203 of the German Criminal Code. The legal requirement for medical confidentiality thus extends beyond the death of the patient, whereas the provisions under data protection law do not. The obligation to observe confidentiality also applies to education research studies in regard to the knowledge acquired about details concerning study participants and staff.
Rec-Protect_2.4<br><br>If images, videos or audio recordings are made of the study participants during data collection, then either pseudonymized or anonymized transcripts should be created or the recordings themselves should be pseudonymized or anonymized.<br>If this is impossible for scientific and/or practical reasons, appropriate data security measures should be taken for analyzing and archiving the images or recordings.<br><br>The study participants should be duly informed about the data processing.	**Definitions:**<br>• *Anonymization* in this context means replacing or modifying attributes which directly or indirectly identify a person, e.g., pixelating faces, blurring or changing names and location information. For the definition of *anonymous data*, see Rec-Protect_1.4.<br>• In the context of images or audio/video recordings, *pseudonymization* can be referred to if it remains possible for the people with access to the decoding list to link the recording or transcript to the data subject via a pseudonym. For the definition of *pseudonym*, see Rec-Protect_1.3. The procedure for pseudonymization corresponds with that for anonymization because in both cases the document or medium is anonymized. The difference is that for pseudonymization a non-semantic identifier is assigned so that authorized individuals can restore the connection to the person as needed.<br>**Reason:** Images and audio/video recordings contain personal data such as, e.g., voice, physical appearance and statements that allow for conclusions to be drawn, directly or indirectly, about the identity of the study participants. This measure contributes to the protection of the study participants and identifies options for legally compliant processing of the collected research data.<br>**General information:**<br>• In cases where neither the images/recordings nor the transcripts are anonymized or pseudonymized at any time during the research process (e.g., because the research question can only be adequately answered by not doing so), an explanation is needed for the people interested in study participation as well as the explicit consent from those who decide to participate in the study. Measures falling within the scope of data protection law must be followed.<br>• When working with images and audio/video recordings, care should be taken that the number of study participants is large enough so that anonymization of the sample and presentation of the results are possible in a publication. If, for methodological reasons, a large number of study participants is not intended, other possibilities should be found to describe the participants' characteristics and present the results in a published paper in such a way that no conclusions can be drawn about individual people.<br>**Possible strategies and suggestions:**<br>• One possible approach to this is to anonymize or pseudonymize the images or audio/video recordings or their transcripts and delete the originals as soon as possible in the research process. This also involves taking the appropriate data protection measures and restricting access to the originals to only those members of the research team who absolutely require it until the originals have been erased.<br>• Deletion of the original recordings is, however, not always part of the plan, e.g., when the sponsoring institution requires that the original recordings be saved for a specific amount of time after study completion. Even when it is necessary to keep the original recordings, it should also be determined if anonymization or pseudonymization of the transcripts or recordings makes sense for the analysis, e.g., to ensure that the researchers are blinded in relation to the participants.<br>• If there is no legal requirement to store the audio/video recordings or images created as part of the study for a specific length of time after study completion, but the researchers, nonetheless, wish to archive the data, it should be determined whether it is sufficient for the purpose of archiving to save the anonymized transcripts and/or recordings once the study is completed.

	• Regardless of the form in which the data collected for the study (raw data or already processed data) are saved or if there is a requirement to store the data after study completion, the data protection measures should be implemented and followed over the entire process and the people interested in study participation should be informed about the process and their rights. It is therefore recommended that it be determined when planning the study if there are legally binding requirements or individual demands to archive the collected data and which aims are associated with this in order to undertake the appropriate measures based on locally applicable law.

3) Protecting the Privacy and Identity of Study Participants and Individuals Who Decide Against or Terminate Participation	
Rec-Protect_3.1<br><br>If unauthorized third parties are present while data is being collected, it should be ensured that these parties do not acquire or gain any knowledge regarding the information given by the study participants in the study.	**Definition:** *Third party* or *unauthorized third party* refers to a natural or legal person, public authority, agency or body other than the data subject, controller, processor and persons who, under the direct authority of the controller or processor, are authorized to process personal data (Art. 4 (10) GDPR).<br>**Reason:** This measure helps ensure that participants can share their information in a safe and discreet environment without fearing that their responses could be disclosed to unauthorized parties.<br>**Notes:**<br>• *Individual data collection via questionnaire:* If, at the time of data collection, several potential study participants are in the same room at the same time separately filling out the survey (online or on paper), only the participants themselves should have direct knowledge of what each of them writes down. Accordingly, care should be taken that the room offers enough space for the questionnaire to be filled out confidentially or that the participants can decide for themselves where they fill out the questionnaire.<br>• *Individual interview:* Only the research team members present during the data collection and the study participant should have direct knowledge of what was verbally stated during the interview. The interviews should be scheduled so that the participants do not encounter each other, and the interviews should be held in a place that protects privacy.<br>• *Group format for data collection:* If, at the time of data collection, there are several study participants and research team members in the same room together and, e.g., a group interview is being held, only the study participants and the research team members should have direct knowledge of what the people present stated verbally. Non-disclosure agreements with the participants can be conducive to preventing the disclosure of confidential information to third parties. Unauthorized third parties in this case are persons who do not participate in the study, e.g., technical staff or random passers-by.<br>• If unauthorized third parties are present during data collection, it should be ensured that these individuals do not acquire any knowledge about non-participation in the study or termination of study participation.

4) Handling Potential Risks and Stress for Study Participants and Individuals Affected by the Study	
Rec-Protect_4.1<br><br>In the planning stage of the study, the research team should identify potential psychological, physical and social risks and stress that could come from study participation and appropriately inform the people interested in participating about these potential risks and stress.	**Definitions:**<br>• The term *psychological stress* refers to the entirety of the influences that affect the participants as a result of their participation in the study and that can have stressful psychological effects, e.g., on the person's experiences and behavior. For example, triggers (e.g., sensitive topics) at the time of data collection can elicit strong emotional reactions.<br>• *Physical stress* refers to the physical effort which can be connected with study participation and, under circumstances, can go beyond the ordinary physical demands of daily life.<br>• *Social risks* mean that participation in a study can influence how the study participants are viewed by others making it possible that their status in a group or their level of social inclusion could change.<br>**Reason:** Early identification of and open communication about potential stresses help to ensure that the decision to participate or not is an informed one and can be made without hidden risks.
Rec-Protect_4.2<br><br>The research team should design the data collection so that the potential psychological and physical stresses identified during study planning are reduced to an acceptable minimum for the study participants. It is possible that stressors not considered during study planning occur during the data collection. The response to these should be appropriate.	**Reason:** Unnecessary stress can be reduced through targeted adjustments in how data is collected which can contribute to protecting the participants' psychological and physical health and creating a safe environment during data collection. An appropriate response to the occurrence of new stressors during data collection (e.g., a strongly emotional reaction during an interview) can reduce the intensity of the stress for the participants, lead to adjustments in future situations where data is collected, and/or raise awareness in the research team about other stressors.<br>**Note:** If patients are present during data collection but no data is collected from them (e.g., in an observational study of bedside teaching), the data collection should not have any negative consequences on further medical treatment.
Rec-Protect_4.3<br><br>The research team should identify which people or groups of people who are considered for study participation could be vulnerable. This is the prerequisite for being able to carefully weigh the participation of vulnerable individuals against the potential risks and stress involved with study participation. Enhanced protective measures should be put into place, as needed, to guarantee safe participation for vulnerable individuals. People for whom the risk of participation is unacceptable should be excluded from study participation in a logical and transparent manner.	**Definition:**<br>In education research studies, the following factors can indicate vulnerable people:<br>• Interdependent relationships between study participants and researchers<br>• Members of marginalized groups, e.g., ethnic minorities or refugees<br>• An elevated risk of harm during study participation due to a physical and/or mental illness or pregnancy<br>• A limited or lack of ability to make an informed and autonomous decision to participate or not.<br>**Example:** In a study investigating, e.g., the connection between sleep deprivation and cognitive performance in medical students, it is possible that the risks of sleep deprivation for certain people, such as those with epilepsy or diabetes, cannot be sufficiently minimized. In such cases, participation would be connected with too high a health risk, which is why it is logical that people with these characteristics (epilepsy, diabetes) should be excluded from participating.<br>**Reason:** This measure helps ensure that potentially vulnerable people are not excluded from study participation per se, but rather only if the risk cannot be reduced to an acceptable level. Furthermore, this measure should result in the research team taking measures to make study participation as safe as possible for potentially vulnerable people.<br>**Notes:**<br>• If vulnerable people participate in the study, additional relevant legal provisions should be checked and the advice of the responsible ethics committee considered in order to ensure the protection and voluntary, informed participation of these people, such as, e.g., consent by a legal guardian if a person is unable to give consent on their own behalf.<br>• Evaluating if and to what extent a person may be vulnerable is in itself not a stigmatization.

	• Attributing vulnerability to a person for purposes of protection with the potential consequence of excluding that person from a study must be weighed against any disadvantage to the person resulting therefrom.
Rec-Protect_4.4<br>The research team should make efforts to avoid disadvantages in regard to all levels of education and training and the future career paths of the affected persons as a result of participating or not participating in the study.	**Example:** A new teaching format is piloted in a required course and scientifically evaluated. Half of the study participants are taught using the new format, and the other half according to the traditional method. It is possible that the group who received instruction through the new method scores higher on exams than the group who were given conventional instruction–or vice versa. A cross-over study design could be applied here, in that during the first phase one half of the students are taught using the new teaching format and the other half with the traditional one. Data collection for the study is then carried out after the first phase. In the second phase, the intervention is switched to the control group, and after that the regular exam or assessment is administered, e.g., a written test in undergraduate or graduate education, professional or post-licensure training.<br>**Reason:** Participating or not participating in an education research study should not negatively influence the course or success of training or education or the career path. To ensure this, appropriate compensation for disadvantages is important.<br>**Note:** Disadvantages, such as lower exam scores that could be earned in connection with study participation, can be compensated for through offering options to catch up on learning content or repeat assessments.
Rec-Protect_4.5<br>Deceptive techniques should only be used after carefully considering if they are necessary to answer the research question.	**Definition**: *Deceptive techniques* refer to methods in which people interested in study participation and study participants are given irrelevant information about the study or information about the study is withheld, e.g., in order to gather unbiased data.<br>**Reason:** Usually, it is ethically unacceptable to deceive people interested in study participation or study participants. However, the use of deceptive tactics can be justified if their use is expected to result in a meaningful gain in knowledge that cannot be achieved any other way. It should be ensured that the physical and/or psychological risks and stress are minimal when using such techniques.<br>**Note:** If deceptive techniques are being used, information should be provided in the informational material about the study and in the interviews stating that not all of the information about the data collection can be shared. Information should also be given stating that after data has been collected a full explanation will be provided and that afterward there will be the opportunity to have the collected data erased.
Rec-Protect_4.6<br>If a member of the research team is present when the data is collected, it should be ensured that this person has an impartial relationship to the study participants. This is particularly important if this person directly witnesses what the participants say or do during data collection.	**Definition**: The term *impartial* is used in this context to mean that the research team member present has no personal or professional relationship to the study participants and is approachable while remaining neutral in their conduct toward the study participants.<br>**Examples of potentially biased individuals when the study participants are students:** Teachers with or without authorization to administer assessments, fellow students, fellow trainees<br>**Reason:** This measure contributes to a safe environment for data collection so that study participants can think freely and express themselves without concern.<br>**Note:** In qualitative interviews it can be advantageous when researchers and participants already know and trust each other. This can lead to an interview situation in which participants are able to express themselves freely. Nevertheless, it can still be helpful to verify prior to data collection that no bias is present. If there is a bias, then another person from the research team should perform the data collection.
Rec-Protect_4.7<br>The research team should identify potentially affected non-participants prior to data collection in order to inform them and, if necessary, take measures to protect their privacy.	**Definition**: *Affected non-participants* are people who do not participate in the data collection but are still affected by it. This means that no data is collected from these people but the collection of data can impact them.<br>**Examples of potentially affected non-participants:** workers in the various healthcare professions, patients and their relatives, minor-aged patients and their relatives, patients lacking legal capacity, teachers, simulated persons.<br>**Examples of possible impacts of data collection on non-participants:** If relatives or visitors are present in a hospital room where the bedside teaching is being filmed for research purposes, these people will be asked to leave the room.<br>**Reason:** If potentially affected non-participants have been identified, they can be informed about the study, unintended data collection can be avoided and, if necessary, additional measures can be taken to protect the rights and privacy of both the study participants and the affected non-participants.

5) Ensuring Informed and Free Decisions about Study Participation	
Rec-Protect_5.1<br><br>The research team should ensure that the information through which people first learn of the study does not raise unrealistic expectations or contain false promises.	**Reason:** People should be made aware of the study in a straightforward manner with the most important information so that, based on it, they can make the decision to take any necessary further steps to participate in the study. Promises can give rise to expectations or exert unwarranted pressure and stand in opposition to the notion of free and informed decisions for or against study participation.<br>**Example:** "Want a better grade on the pharma exam? Participate in our study!" This headline can seem to say that participating in the study is connected with better grades in pharmacology, although this cannot be guaranteed.
Rec-Protect_5.2<br><br>If study participants receive recognition for their participation, this should be listed in the informational letter and designed such that the free decision for or against study participation is not influenced.	**Definition:** Here *recognition* is understood to be a token of appreciation for the time and effort put forth by the participants, such as, e.g., a gift card for a small amount. Such recognition makes it possible to renumerate the expense of participation without applying pressure to participate.<br>**Reason:** Too generous or inappropriate recognition can undermine the free nature of the decision in that it exerts pressure on potential participants or acts as an incentive that overcompensates for the risks and effort of participation. This measure can reduce the risk that people feel pressured to participate in the study for the recognition and, as a result, are less able to make an independent decision.<br>**Notes:**<br>• The recognition should be distributed fairly and transparently among the study participants, in that, e.g., every person receives recognition or a lottery is held.<br>• If the study participants involve undergraduate/graduate students or vocational trainees, the recognition should not have any influence on education, training or career path.<br>• Covering travel costs to the site of data collection can likewise be relevant but is, however, not viewed as recognition for study participation but rather as a reimbursement of expenses.
Rec-Protect_5.3<br><br>The research team should ensure that terminating study participation is possible at any time without negative consequences and that the people interested in study participation are explicitly informed of this.	**Reason:** This measure has three components: 1) ensuring the option to terminate study participation at any time, 2) guaranteeing that terminating study participation does not have any negative consequences on education, training or career path, and 3) communicating these conditions to the people interested in study participation. This strengthens the basis for free participation and prevents the participants from feeling obliged to continue on through to the end of the study.<br>**Notes:**<br>• If patients are participants in an education research study, it should be ensured that terminating participation does not have any negative effects on further medical care. This should also be ensured when patients are present for data collection but no data is collected from them. For example, if in an observational study of bedside teaching situations, the study participants, e.g., medical or nursing students, terminate their participation.<br>• In the informational materials about the study, it should be pointed out that anonymous or anonymized data which was already collected before terminating study participation can no longer be erased.

Rec-Protect_5.4<br><br>The research team should write an informational letter containing all of the relevant details about the study to be given to the people interested in study participation.	**Content of an informational letter:**<br>• Purpose and aim of the study<br>• Study procedure and duration<br>• Inclusion and exclusion criteria for study participation<br>• For interventional studies: methods for assigning participants to the experimental and control groups<br>• Method and type of data collection and analysis<br>• Potential risks and stress arising from participation<br>• Potential benefits of participation<br>• Information about privacy and data protection<br>• Information about the procedure to request the erasure of research data<br>• If applicable, information about recognition and/or reimbursement for participating<br>• If applicable, information about insurance coverage for the participants (e.g., insurance for traveling to and from the study site)<br>• Person to contact in the case of questions<br>**Reason:** People who are interested in study participation should, as a result of the informational letter, be in a position to assess the potential risks and stress associated with study participation and to weigh them up against the benefits of participation. They should also be able to assess their potential suitability or aptitude for study participation. Based on the informational letter, these people should be able to decide if they want to sign up for an informational interview with the research team or, when no consultation is needed for study participation, if they want to participate in the data collection.<br>**Notes**:<br>• Additional information could be important for the letter that is not mentioned in the list above.<br>• It should be documented which information from the above list is not included in the informational letter and why.<br>• The informational letter should be written with the target audience in mind. For minors or individuals with a limited or lack of ability to give consent, an adapted version of the informational letter or another mode of communication may be necessary if these groups come into question as study participants.
Rec-Protect_5.5<br><br>If personal and/or sensitive personal data will be collected, the research team must write a data protection declaration and/or informational materials about the data processing that comply with the applicable data protection requirements. This declaration must be available to the people interested in study participation and discussed in the informational interviews, if any are held.<br><br>If data processing is not based on consent but rather on other legal grounds, the duty to inform about the data processing also applies if personal data is processed (meaning directly identifying or pseudonymized data).	**Content of the data protection declaration / Information about data processing**<br>• Name and contact information of the responsible person (e.g., the institution(s) that carry out research projects and the study directors there)<br>• If applicable, the name and contact information of the data protection officer<br>• Purpose of the data processing<br>• Type of data collected<br>• Legal basis (generally this is the "consent of the data subject"; to the extent that processing is done on the basis of Art. 6 (1)(f) GDPR, justification of "legitimate interest")<br>• If applicable, data transfer / recipient of the data<br>• If applicable, intention to transfer data to a third country or international organization, absence or presence of an adequacy decision and any guarantees to protect data<br>• Duration of data storage or criteria for determining the duration<br>• Rights of the data subject (information, rectification, erasure, etc.)<br>• Information about the right to object when data processing is based on consent<br>• Right of appeal to the supervisory authority<br>**Reason:** The data protection declaration is meant to place individuals in a position to make an informed decision on the basis of the information contained therein about whether or not they wish to share their data. It is supposed to create transparency and ensure that the people interested in study participation and study participants understand their rights and know how their data will be used and protected. The information about data processing and its content is laid down in Art. 13 and Art. 14 GDPR.<br>**Notes:**<br>• Additional information could be important for the data protection declaration that is not mentioned in the list above.

	• Ethics committees often provide templates for data protection declarations which should be used to ensure legal validity.<br>• If non-personal data will be gathered in the study, the researchers should still describe in an informational letter how data will be handled responsibly and protected against third-party access, e.g., in the case of an anonymous online survey.
Rec-Protect_5.6<br><br>The research team should ensure that the declaration of consent for study participation<br>• complies with the legal requirements,<br>• is available in the proper form for all groups of people who come into question for study participation,<br>• corresponds to the form and content of the data collection, and<br>• is given voluntarily and in an informed manner.	**Definition:** *Declaration of consent* refers to the act through which people indicate that they want to participate in a study. The prerequisite for study participation is consent that is given freely and in an informed manner. *Informed* means that the person interested in study participation has read and understood the informational letter. *Free* means that no pressure or force was exerted on the person interested in study participation in order to get them to consent to participate (see Art. 7 and Art. 9 (2)(a) GDPR).<br>**Examples of the forms for declaring consent:** Signing a declaration of consent, verbally affirming participation, or an opt-in choice for online data collection.<br>**Reason:** The measure helps ensure that the declaration of consent meets the requirements and is appropriate to the study.<br>**Notes:**<br>• For minors who are interested in study participation, both the minor and their legal guardian(s) should give consent to participate in the study. The wording of the consent form should be adapted, as needed, for the particular age group. It should also be determined if additional rules must be followed regarding interactions with minors in the research context.<br>• People unable to give consent on their own behalf and for whom study participation is potentially possible: Consent should be declared by the legal guardian or custodian. The person unable to give consent should be given the opportunity to communicate whether or not they wish to participate in the study. This can be done, e.g., through posture, facial expression, and verbal statements, and these should also be watched for and noted during data collection. Furthermore, it should be determined if additional rules must be followed regarding interactions in the research context with individuals who are unable to give consent.<br>• People whose ability to consent is questionable and who are interested in study participation: If the impression exists that a person is unable or only limitedly able to give consent, e.g., due to mental strain under exceptional circumstances, the opportunity to consent to or decline study participation can take place at a later point in time. Implementing other measures could also make sense to enable these individuals to give their free and informed consent.<br>• If natural research data, e.g., portfolios or essays written as part of exams, will be gathered and analyzed for the study, this can require the consent of the authors. This can also be the case with secondary data analysis. The conditions under which natural research data and secondary data can be used should be verified specifically and implemented accordingly.
Rec-Protect_5.7<br>The research team should hold personal interviews with people who are interested in study participation if this is required for participation.	**Definition**: A personal interview is a direct interaction between an authorized member of the research team and a person interested in study participation.<br>**Reason:** A personal interview is required for certain studies to ensure that those interested in participation have fully understood the informational letter and the declaration of consent/information about data processing and are able to make an informed decision (*informed consent*) to participate or not to participate. Especially in the case of complex studies that are connected with risks or to which specific ethical and/or data protection rules apply, personal interviews enable clarification of all open questions and assure that the study and its effects are fully understood.<br>**Notes:**<br>• Whether or not a personal interview is required for participation should be considered when planning the study. When doing so, the nature of the study and the legal requirements should be considered.<br>• Interviews with minors who are interested in study participation: The interview should be held with the minor and their legal guardian(s). The structure and course of the conversation should be linguistically adapted, as needed, for the age group in question to ensure a free and informed decision for or against study participation. It should also be determined if additional rules must be followed regarding interactions with minors in the research context.<br>• Interviews with people unable to give consent on their own behalf and for whom study participation is potentially possible: The interview should be held with the legal guardian or custodian. The attempt should be made to inform the incapacitated person to the best extent possible. It should also be determined if additional rules must be followed regarding interactions in the research context with individuals who are unable to give their consent.

Rec-Protect_5.8<br>If no personal interview is required for participation in the study, the research team should still ensure that all of the questions asked by people interested in study participation have been answered appropriately.	**Reason:** Clarifying any open questions will ensure that the conditions for study participation have been understood and an informed decision can be made.<br>**Note:** At least one person from the research team should be responsible for answering questions and their contact information should be listed in the informational letter. A deputy for the responsible person should also be designated.
Rec-Protect_5.9<br>If a written or verbal declaration of consent is required, the research team should ensure that people have a sufficient amount of time to consider their decision for or against study participation.	**Reason:** This measure is conducive to reaching decisions for or against study participation without time pressure. The time for deliberation allows a person interested in participating to read the informational letter once again, clarify open questions, and arrive at an informed decision.<br>**Note:** The time allowed for consideration should be appropriately proportionate to the potential risks and stress of study participation. Recommendations regarding this commonly cite 48 hours as a guide. However, it should be ascertained if this amount of time fits well with the planned study.
Rec-Protect_5.10<br>If a member of the research team has<br>• drawn someone's attention to the study and/or<br>• held an interview and/or<br>• is present when the written consent form is signed,<br>it should be ensured that this person is impartial and unbiased in relation to the person being informed or the person interested in study participation.	**Definition:** In this context, *impartial* and *unbiased* are understood to mean that the research team member who is present has no personal or professional relationship to the person interested in study participation and that the team member is attentive but unbiased in their behavior toward this person.<br>**Example:** A person is a teacher and a member of the education research team. A study is being conducted for which individuals with whom the research team member has contact as a teacher are eligible. If the team member with the dual role (teacher and researcher) is present in the situations described here, it is possible that the people interested in study participation might feel pressured to participate because they assume there will be negative consequences if they do not.<br>**Reason:** The neutrality of the individuals from the research team helps to reduce the risk of influencing a decision for or against study participation due to power relationships, personal relationships or dependencies. This contributes to protecting the autonomy of the potential participant and facilitates a decision for or against participation based on a person's own conclusions and not on external expectations or obligations.<br>**Note:** If it is not possible for a person with no personal or professional relationship to the person interested in study participation to be present in the situations described here, the individuals involved should consider if an impartial interaction is possible.
Rec-Protect_5.11<br>The research team should keep information about study participation and non-participation confidential. Any person interested in study participation should be explicitly informed about this obligation to observe confidentiality.	**Reason:** This measure has two basic components: 1) the obligation of the research team to treat the information pertaining to this measure confidential, and 2) informing the people interested in study participation about this confidentiality. The confidential handling of this information helps ensure that the participants can independently decide whether or not to participate and if and how they communicate their decision to the people around them. This can help prevent any negative social reactions to a person's participation or non-participation in a study.

6) Assuring Independent Ethical Review and Legal Compliance in Study Planning and Conduction	
Rec-Protect_6.1<br><br>The research team should submit the study protocol to the responsible ethics committee for review.	**Definition:** An ethics committee is an independent body that evaluates the ethical and legal aspects of an intended research study, along with its appropriateness, according to the most objective criteria possible and discusses it with the researchers, e.g., in the form of peer-to-peer consultation.<br>**Reason:** Education research studies in the health professions usually pose minor risks of harm to the study participants. Nonetheless, an independent evaluation can help ensure that the study is ethically defensible. Regardless of whether or not the advice of an ethics committee is required (e.g., by a professional code of conduct) or voluntary, an independent review of the intended study is usually valuable. Moreover, scientific journals often require authors to submit proof of ethical approval when publishing original papers on studies in humans.<br>**Notes:**<br>• Whether or not an ethics committee must be consulted depends on several factors, e.g., the country, federal state or canton in which the study will be conducted, the type of study, the study participants, the researchers (e.g., members of the medical board or association) and the institutional or university rules and regulations.<br>• There may be no access to an ethics committee. Nevertheless, detailed documentation should be compiled on how ethical research and legal aspects were taken into account. Also, it can help to consult with experienced colleagues who are not involved in the study.
Rec-Protect_6.2<br><br>When planning, conducting and publishing a study, the research team should ensure compliance with the relevant local, regional and national laws and regulations.	**Definition**: *Relevant laws and regulations* refer to all of the legislation that must be observed when conducting the study and serves to protect the study participants.<br>**Examples**: Legal regulations and provisions governing employment, maternal leave, and the protection of minors; professional codes of conduct<br>**Reason:** Adhering to the applicable laws and regulations contributes to the protection of the study participants and the individuals potentially affected by the study.
