# Supplementary material for: Developing recommendations on ethical aspects affecting studies in health professions education research
Source: GMS J Med Educ. 2026 Jan 15;43(1):Doc1. doi: 10.3205/zma001795 (PMC12875059; doi:10.3205/zma001795)
Supplement: Recommendations on assuring the quality of a study and its publication (rec-quality) [file JME-43-1-s-004.pdf]

Attachment 4: Recommendations on assuring the quality of a study and its publication (rec-quality)

Table: Legend for the research process icons

Icon	Stage in the research process
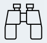	Survey of topics & state of research
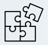	Identification of a research gap
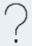	Formulation of a research question
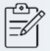	Study planning
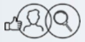	Recruitment
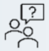	Data collection
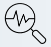	Data analysis
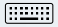	Writing the paper
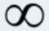	Measure affects the entire research process

Note

The recommendations should be understood to supplement existing guidelines and codes of conduct. They are based on the local perspectives of the authors (DE, CH, AT). Users should check the rules and regulations applying to them and adapt these recommended measures accordingly.

Explanations

Each recommended measure has been assigned an identifier. The logic behind these identifiers is as follows:
Short version of title_area number.measure number
Icons have been created to give a clear overview of the time points at which each measure is relevant in the research process.

1) Assuring Good Scientific Practice

Rec-Quality_1.1

The research team should ensure that the study focuses on a relevant research question that has been developed based on recognized scientific principles. This means that the question has been derived from an identified gap in the research.

Definitions:

- *Relevant research question:* The research question identifies the area of investigation. The relevance of the research question can lie in the expectation that answering the research question will contribute to the body of knowledge in health professions education research or to improving undergraduate and post-graduate education and training and advanced professional training in these professions. The research question should be formulated before the methods are defined and data collection starts.
- *Recognized scientific principles:* The research team should develop the research question based on a gap in the research which has been identified in the results and findings of existing studies and theories on the topic. Before a research question can be formulated, the state of research should be ascertained by means of an extensive review of available studies, literature and data regarding the topic. The available studies should be critically evaluated in terms of their currency, methodology, relevance and scientific validity. The currency of a study should not be determined solely by its publication date since there are studies whose findings cannot be refuted even years afterward.

Reason: Adhering to recognized scientific principles forms the basis for identifying a research gap and deriving from it a relevant research question. Asking a relevant research question enables targeted data collection and analysis which, on the one hand, leads to reproducible results and, on the other, avoids wasting resources.

Notes:

- The term "research question" is used here in the singular; however, it is possible for one study to answer several research questions.
- If phenomena are by chance observed in a study, e.g., through the analysis of confounding variables (quantitative research) or through other aspects that are mentioned in interviews (qualitative research), these phenomena can be investigated in a new study. These incidentally discovered phenomena should not be presented as the results of the original study because it was not designed to investigate these phenomena and other research methods may have been necessary to do so.
- More information on the principles of good scientific practice can be found, e.g., in the German Research Foundation's (DFG) *Guidelines for Safeguarding Good Research Practice*.

Rec-Quality_1.2

If the research approach allows, the research team should create a study protocol before data collection begins, and document and justify any deviations from the protocol during the study.

Definition: Here, a *study protocol* is understood to be a written document in which the basic elements of a study are defined prior to study begin. The protocol contains, e.g., background information on the subject, the gap in the research with the resulting research question, the methods for collecting and analyzing data, and information on the intention to publish the results.

Reason: The study protocol is a central part of planning the study and serves several important purposes. First, it can help to systematically define the study procedure in detail, which can improve the quality of study conduction. Second, it serves as a record of any deviations from the procedure as originally planned through amendments to the original study protocol and can thus contribute to the study's transparency and confirmability. Third, the protocol can be used as a basis for evaluations of the study by independent bodies, such as ethics committees. Clear and exact documentation can also help provide precise answers based on facts to questions asked by experts during the peer review process and/or by readers. Thus, processes and procedures do not need to be reconstructed from memory, which can potentially result in erroneous information.

Notes:

- Once study planning is complete, publishing the study protocol should be considered, e.g., in a trials register. This makes it possible, once the results are published, to see where deviations from the original plan occurred. For studies with a quantitative research approach, the risk of p-hacking and HARKing sinks, (*p-hacking:* presenting significant results when no significance actually exists; *HARKing:* presenting a post-hoc hypothesis as if it were an a priori hypothesis).
- If no suitable study register exists or it is not possible to publish the study protocol, the study protocol can be lodged with a neutral and trustworthy individual or institution not involved with the study. This individual or institution should be in a position to safeguard the protocol and ensure that it can be reviewed as needed. The main reasons for lodging the protocol is to ensure transparency and enable external verification of the consistency between the protocol and the actual conduction of the study.

	• For qualitative and participative research, creating and publishing a complete study protocol before data collection begins is not necessarily meaningful. When pursuing these research approaches, it is usually impossible to define all of the aspects in advance, instead the study design is often adapted during the course of the study. Therefore, for this type of research the focus is on continual and ongoing documentation and justification of the approach.
Rec-Quality_1.3<br><br>When planning, conducting and analyzing the study, the research team should follow recognized methodological approaches that are suitable for answering the research question. Since doing this is not always possible or does not always make sense, please refer to the note regarding this measure.	**Definitions:**<br>• *Recognized methodological approaches:* Belonging to these are established study designs and procedures for selecting study participants, taking samples, and collecting and analyzing data. Such recognized and accepted approaches are described in textbooks and reference books and documented in the methods section of published original works. The scope of *recognized methodological approaches* also includes the use of data collection tools, about which it can be assumed that their use enables the collection of the data needed to answer the research question. For quantitative data collection, reliable and, if possible, validated tools should be used if they are available. For qualitative data collection, the tools should be developed in a justified manner grounded in theory.<br>• *Methodological approaches suitable for answering the research question:* This means that the methodology should be chosen such that the research question can be answered with the highest degree of precision and reliability possible. The selection of the methodological approach will therefore be determined by the research question.<br>**Reason:** This measure helps ensure that the study is planned and carried out in such a way that the research question can be answered with reliable and, where possible, reproducible data. This minimizes the risk of methodological errors, ensures efficient use of resources, and helps ensure that the time spent by the participants and the gathered data serve a meaningful purpose. Furthermore, this measure forms a basis on which study results can yield additional value and benefit.<br>**Note:** If there are no recognized approaches for specific methodological aspects or there are compelling reasons to deviate from recognized approaches, the research team may develop its own method which should be based to the extent possible on existing (epistemological) theoretical concepts. These approaches and their corresponding theoretical considerations should be documented and presented as completely as possible in the publication in order to ensure verifiability and, if applicable, reproducibility of the results. In principle, the development of methods represents a separate area of research that requires clear delineation from the application of the methods.
Rec-Quality_1.4<br><br>It should be ensured that the skills and expertise in the subject matter and methodology required for the study are present in the research team. This means that the various competencies are collectively present and not that each member must possess all of the skills and expertise needed.	**Definitions:**<br>• *Subject matter competency* is understood to mean that the people working on the study are able to contribute substantially to the study by ascertaining the current state of research, deriving the research question from it and/or interpreting the results.<br>• *Methodological competency* is understood to mean that the people involved in data collection and analysis have practical and theoretical knowledge of the methods used to answer the research question.<br>**Example:** To investigate the influence of group dynamics in interprofessional teams on patient safety using group interviews, the research team should have members with well-founded expertise in group dynamics, interprofessionalism and patient safety, as well as members with theoretical knowledge and practical experience in developing, conducting and analyzing group interviews.<br>**Reason:** This measure helps to ensure that the study is carried out according to a high standard and that it can provide a significant contribution to the state of knowledge in health professions education research and/or the quality assurance of vocational training, undergraduate and postgraduate education, and advanced professional training in these professions.<br>**Note:** If not all of the methodological or subject matter competencies are covered by the research team, this can be remedied by drawing on external expertise, e.g., through consultations and, if applicable, by adding people with the needed expertise to the research team. Furthermore, "safeguarding" can entail additional steps such as, e.g., training the individuals involved for data collection and/or analysis, as well as guiding and supervising inexperienced co-workers.

Rec-Quality_1.5<br><br>The research team should check for any conflicts of interest in the collaborators and, if needed, take the appropriate measures.	**Definition:** A conflict of interest exists when the primary interest of the researchers to objectively investigate the object of study and present the results without bias could be compromised by secondary interests.<br>**Potential secondary interests that could conflict with the primary research interest can include:**<br>• Financial connections to businesses which could profit from the study results;<br>• Intellectual conflicts of interests, e.g., in individuals whose theoretical convictions can compromise objectivity;<br>• Participation of a person in the decisions made by a committee of a professional association or a scientific society to which the effects and influences are to be investigated in the study in which the person is involved.<br>**Reason:** This measure helps recognize conflicts of interest early on and reduces the risk of conscious or unconscious misconduct due to a conflict of interest.<br>**Note:** When there are conflicts of interest within the research team, consideration should be given to deploying the individuals in question to parts of the study where the conflict of interest has no influence or, failing that, excluding the individuals from working on the study. In cases of less serious or potential conflicts of interest, openly identifying the conflict in front of the collaborators and ethics committee and disclosing it in publications can suffice.
Rec-Quality_1.6<br><br>The research team should reflect on what diversity means in their particular study and how it can be taken into account appropriately. This entails identifying the aspects of diversity relevant to the specific research question and implementing them in a sensitive manner without the need to include all groups of people, characteristics and traits in every study.	**Definition:** What is understood here by "diversity" is the respectful recognition and furtherance of human diversity in the scientific context. This refers to both the study participants and the research team itself.<br>**Example:** Assuming that anesthesiology as a specialty is becoming more and more popular among female physicians, a study seeks to investigate the experiences of female medical students who have spent part of their medical residency in anesthesiology. In this case, diversity does not mean that male medical residents should be included in the study. Instead, diversity could lie, e.g., in considering different age groups, ethnic backgrounds, social or economic conditions, prior experiences in medicine, or different universities. These factors could significantly influence the participants' experiences and contribute to a differentiated examination of the results.<br>**Reason:** Deliberately asking what diversity means in the context of a particular study can promote an inclusive research practice.
Rec-Quality_1.7<br><br>If data on groups of people is to be collected at a specific site, the research team should check if these groups of people are already participating in multiple ongoing education research studies at that site and if a study on a similar topic is being conducted there.	**Reason:** This measure can help protect groups of people from receiving a large number of study requests and make it more likely that an adequate number of cases is attained.<br>**Notes:**<br>• Institutional entities which could provide information on current data collection are, e.g., the clearinghouse for academic teaching projects, research coordination offices at universities or other institutions, curricular development offices, and offices involved in evaluations and quality management.<br>• If this information cannot be gathered because, e.g., there is no central coordination of ongoing studies, the research team can still respond to the feedback from the people interested in study participation and/or study participants and make adjustments, as needed, to recruitment or communication.

2) Complying with Legal Requirements and Standard Guidelines	
Rec-Quality_2.1<br><br>The research team should inform themselves about the rules, regulations and statutes applicable to the study and comply with them.	**Definition:** Rules, regulations and statutes refer to all of the legal requirements relevant to planning and conducting a study.<br>**Examples:** Rules for course evaluations in higher education, rules for examinations and assessments, other internal rules, such as those for using social media platforms.<br>**Reason:** It is impossible to conduct a study without complying with the applicable legal requirements.
Rec-Quality_2.2<br><br>The research team should ascertain which committees should be involved in regard to conducting the study and consult with them accordingly.	**Definition:** Committees in this context refer to individuals and groups who have certain responsibilities within an institution and could be relevant to carrying out a study.<br>**Examples:** Staff council/works council/employee representative body, university or institutional leadership, corporate communications team, student council<br>**Reason:** As a result of this measure, legitimate interests and concerns are taken into account during the planning and conduction of the study. In addition, the involvement or approval of the responsible committees may be a prerequisite for conducting the study.

3) Visibility of the Study	
Rec-Quality_3.1<br><br>The research team should report on the study.	**Definition:** Here, reporting on the study means publishing the background information, methods, results and a discussion of the study in such a manner that this is available to the target group, meaning those for whom the study results could be significant, and other interested parties. Publication can be in professional journals, public databases or via other suitable channels, and supplemented through conference presentations.<br>**Reason:** This measure helps ensure that scholars in the field can inform themselves about the current state of research and/or that the results can be applied at all levels of education and training and/or that the results can provide support for decisions on structural changes in research or education in the health sciences.<br>**Notes:**<br>• Regardless of whether or not the results of a methodologically rigorous study correspond with the expected findings, these belong to the current body of knowledge and should be made available to the public. This also avoids publication bias.<br>• It is recommended that the paper be submitted to a peer-reviewed journal since this can assure the quality of the paper. *"Predatory scientific publishers"* should be avoided because they often apply inadequate quality standards to published texts and lack transparency in how they work. Both of these issues can reduce the study's credibility.<br>• Consideration can be given to making the information, materials and data from the study available via a suitable repository, archive or in an attachment to the published paper, and in accordance with FAIR Guiding Principles (Findable, Accessible, Interoperable, Reusable). Alternatively, consideration can be given to keeping the information, materials and data from the study in a manner compliant with data protection law so that it is possible to answer additional questions even after study completion and to allow access in the case of serious and credible inquiries.<br>• When making information, materials and data from a study available, attention should be paid so that no personal data is disclosed. Particularly in the case of qualitative studies, the anonymization of the data should be verified to ascertain the extent to which re-identification is possible. However, this risk should also be noted for quantitative studies because rare and/or individual attributes and combinations of multiple attributes can make re-identification possible.<br>• When publishing the results of a qualitative study in which a single case was analyzed, additional measures should be taken to protect the identity and privacy of the study subject or a more general form should be found in which to present the results.

4) Confirmability of the Study	
Rec-Quality_4.1<br><br>The authors should compose the paper in such a way that readers can achieve a comprehensive overview of the study.	**Examples of a paper's content that aims to provide a comprehensive picture:**<br>• Clear and structured outline, e.g., based on journal requirements or a suitable reporting guideline<br>• Background information on the topic<br>• Current state of research<br>• Research question and justification of why its answer is relevant<br>• Description of the methodological approach, information about any use of previously published data (secondary analysis) and justification for excluding data (e.g., outliers)<br>• Complete and unbiased presentation and description of the results without interpretation by the authors<br>• Discussion of the results and positioning of them in the current research, identification of the study's limitations, discussion of contradictory results within the study and/or in comparison with other studies<br>• Conflicts of interest in those who worked on the study<br>**Reason:** This measure can help facilitate the confirmability of the study report and contribute to the study's reproducibility in the case of a quantitative research methodology. A comprehensive report enables readers to form judgments about the quality of the study in such a way that avoids misinterpretations.<br>**Notes:**<br>• Describing the results in an unbiased and undistorted manner is also part of presenting a comprehensive report on the study.<br>• If the results are to be presented in several publications, it should be checked to see if this approach is appropriate. For instance, if the data to answer two different research questions are collected from surveying the same target group and, for reasons of economy, with one questionnaire, then a separate publication can be justifiable.
Rec-Quality_4.2<br><br>Only those individuals who have made a substantial contribution to the study should be listed as the paper's authors.	**Definition:** Here, a substantial contribution means significant scientific participation in the development of the research question, involvement in data collection, data analysis and/or writing the paper (see, e.g., the German Research Foundation's (DFG) *Guidelines for Safeguarding Good Research Practice* and the recommendations of the *International Committee of Medical Journal Editors* (ICMJE).<br>**Reason:** Individuals who have made a substantial contribution to the study and/or to the publication should be recognized for their work through authorship.<br>**Notes:**<br>• The substantial contributions of each author could be listed, e.g., in a cover letter and, if appropriate, in the paper itself. Identifying the authors' contributions makes the specific role and work of each individual transparent. If doing this is not possible or uncommon in the selected publication format, then documentation of each author's substantial contribution should be recorded and kept.<br>• The authors should mention any individuals who did not contribute substantially but supported the conduction of the study (e.g., academic assistants, institutional leadership, consultants and gatekeepers) in the paper's acknowledgements.<br>• Early and open communication can help clarify the responsibilities of the authors, as well as promote collaboration and avoid conflicts.
Rec-Quality_4.3<br><br>The authors should mark all text passages and figures in the paper that directly or indirectly contain citations from other sources and give clear references to these sources in the bibliography.	**Reason:** Identifying the intellectual property of other authors and marking citations from their publications, along with adhering to journal guidelines and ensuring easy confirmability of the sources are essential aspects of academic integrity and transparency in publications.<br>**Notes:**<br>• The identification of sources extends to both published and unpublished sources, as well as to material from content creators on social media.<br>• A uniform style should be used for citations; this is usually prescribed by the professional journal.

Rec-Quality_4.4<br><br>The authors should list all of the computer software that was used in the study to perform basic steps in the research process.	**Definition:** *Basic steps in the research process* include all of the central activities necessary to plan, conduct, analyze and report on a scientific study. Belonging to these are the identification of research gaps, development of hypotheses, and the analysis and interpretation of the results. Various software programs, such as statistics programs, textual analysis tools or AI-based applications, can be used to assist in performing these activities.<br>**Reason:** Listing these is conducive to creating transparency since it gives readers a comprehensive understanding of how the study results have been obtained. As a result, this supports well-founded evaluations and the reproducibility of the study finding.<br>**Note:** The publication should include information about the software program itself and describe the way in which it was used.
